# Sodium salicylate improves detection of amplitude-modulated sound in mice

**DOI:** 10.1016/j.isci.2024.109691

**Published:** 2024-04-09

**Authors:** Maurits M. van den Berg, Aaron B. Wong, Ghais Houtak, Ross S. Williamson, J. Gerard G. Borst

**Affiliations:** 1 Department of Neuroscience, Erasmus MC, University Medical Center Rotterdam, NL-3015 GD Rotterdam, the Netherlands; 2 Pittsburgh Hearing Research Center, Department of Otolaryngology, University of Pittsburgh, Pittsburgh, PA 15213, USA

**Keywords:** Cellular neuroscience, Sensory neuroscience, Cell biology

## Abstract

Salicylate is commonly used to induce tinnitus in animals, but its underlying mechanism of action is still debated. We therefore tested its effects on the firing properties of neurons in the mouse inferior colliculus (IC). Salicylate induced a large decrease in the spontaneous activity and an increase of ∼20 dB SPL in the minimum threshold of single units. In response to sinusoidally modulated noise (SAM noise) single units showed both an increase in phase locking and improved rate coding. Mice also became better at detecting amplitude modulations, and a simple threshold model based on the IC population response could reproduce this improvement. The responses to dynamic random chords (DRCs) suggested that the improved AM encoding was due to a linearization of the cochlear output, resulting in larger contrasts during SAM noise. These effects of salicylate are not consistent with the presence of tinnitus, but should be taken into account when studying hyperacusis.

## Highlights

• Salicylate reduced the spontaneous activity of units in the inferior colliculus (IC)
• Salicylate improved the encoding of sinusoidally modulated (SAM) noise in the IC
• Salicylate also improved the behavioral detection of SAM noise
• These effects of salicylate may be due to loss of compression in the cochlea

## Graphical abstract

**Figure:**
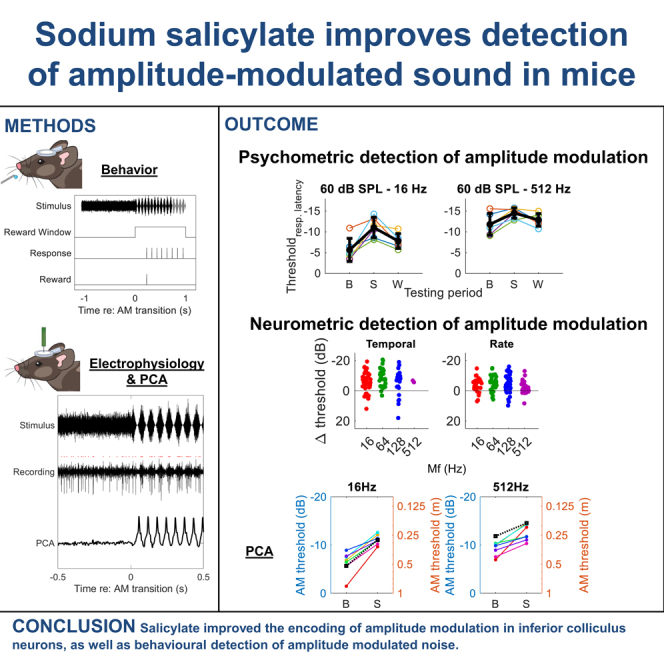

## Introduction

Salicylate, an active metabolite of aspirin, is an ototoxic drug, which induces tinnitus and causes an increase in hearing thresholds.^1^ In humans, both the severity of the hearing loss and the perceived intensity of the tinnitus are correlated to the plasma salicylate concentration.^2^ Salicylate has also become a popular method to study tinnitus in animals.^3^ It has the great advantage that most of its effects on the auditory system are both rapid and reversible, which makes it possible to investigate the changes accompanying tinnitus in great detail.^4^ This contrasts with other methods to induce tinnitus, such as sound overexposure, which are largely non-reversible, and whose time course of tinnitus induction is more uncertain. How salicylate induces tinnitus is still debated. A complicating factor in elucidating its mode of action is that salicylate has multiple effects on audition, both within the cochlea and in the central auditory pathway.^5^

Within the cochlea, salicylate reduces vibrations of the basilar membrane and the organ of Corti; it abolishes the characteristic compressive nonlinearity, linearizing the relation between sound level and vibration amplitudes of the basilar membrane.^6^^,^^7^^,^^8^ This linearization was not observed within the organ of Corti itself, but the best frequency peak is abolished there as well.^6^ These main effects on the cochlea are probably due to a reduction of the electromotility of outer hair cells.^9^^,^^10^^,^^11^

It is generally believed that the central effects of salicylate are more important for tinnitus induction than its effects on the cochlea.^5^ For example, despite a reduction of cochlear output, salicylate enhances sound-evoked responses at the level of the inferior colliculus (IC) or auditory cortex (AC).^12^^,^^13^^,^^14^ A strong argument that salicylate induces tinnitus by an effect on the central auditory system rather than on the cochlea is that the hyperexcitability can also be observed upon local application in the central auditory system, both in the IC and in the AC.^13^^,^^15^

Salicylate also induces a tonotopic reorganization both in the IC and the AC involving a shift of the characteristic frequency (CF) toward the mid-frequency range (about 16 kHz in the rat), the presumed salicylate-induced tinnitus pitch.^12^^,^^16^ In addition, the spontaneous activity of auditory neurons changes following salicylate administration. Both increases in the spontaneous activity rate and increases in burst-type firing have been observed at different levels of the auditory system,^17^^,^^18^^,^^19^^,^^20^^,^^21^^,^^22^^,^^23^ but decreases in spontaneous activity have also been reported.^24^^,^^25^^,^^26^^,^^27^^,^^28^

At the synaptic level, several mechanisms have been proposed to explain how salicylate induces hyperexcitability and tinnitus. Both pharmacological experiments and direct recordings of GABAergic transmission have produced evidence that GABAergic transmission is reduced.^13^^,^^15^^,^^29^^,^^30^^,^^31^^,^^32^ The resulting shift in the balance between excitation and inhibition is thought to result in tinnitus.^4^ Additionally, salicylate may affect glutamatergic, serotonergic, and nitric oxide transmission.^19^^,^^33^^,^^34^^,^^35^^,^^36^

Previous work in humans has shown that salicylate affects the perception of sounds including speech understanding and temporal detection; these changes are thought to be at least partially different from its well-known effects on hearing threshold and the induction of tinnitus.^1^

To better understand the complex effects of salicylate in the central auditory system, and to better delineate effects on tinnitus and hearing threshold from its other actions, we used multielectrode recordings to study the effect of salicylate on spontaneous and sound-evoked activity in the mouse IC. The IC is likely involved in both the generation and maintenance of tinnitus.^37^ The effects of salicylate on the auditory system have mainly been studied in animals other than the mouse. Nonetheless, the mouse has several potential advantages, including the good accessibility of its IC and the availability of genetic models.

We used three different stimuli to investigate how salicylate affects the relationship between sound intensity and neuronal firing. First, we measured frequency response areas (FRAs), which allowed us to detect changes in CF, minimum threshold (MT), and rate-intensity curves. Second, we used dynamic random chords (DRCs), a complex, multitone stimulus that allows to extract a spectrotemporal receptive field (STRF) characterizing how units integrate this stimulus as a function of time, intensity, and sound frequency.^38^^,^^39^^,^^40^ In our analysis, we took local acoustic context into account to look at the interactions between sound frequencies as well.^41^ The STRF allowed us to compare how linear the responses of individual units are in the presence and absence of salicylate.

Third, we used sinusoidally amplitude-modulated (SAM) noise stimuli, as detecting amplitude modulations is very important for the analysis of complex sound stimuli such as speech,^42^ and tuning to AM stimuli often emerges at the level of the IC.^43^^,^^44^^,^^45^

Furthermore, to test the behavioral relevance of salicylate’s effects on IC neurons, we trained animals in an operant conditioning task to detect a transition from unmodulated (UM) noise to SAM noise and assessed their performance after administration of salicylate.^46^^,^^47^^,^^48^^,^^49^

The IC displays an impressive heterogeneity in its neuronal responses, which is evident from spontaneous activity rates, which are presumably inherited from the auditory nerve, but especially in the neuronal tuning properties.^50^^,^^51^ This heterogeneity complicates studying the effects of salicylate on spontaneous activity or neuronal tuning within the IC. We, therefore, made long-lasting multielectrode recordings from the IC and longitudinal recordings using two-photon imaging in mice, which allowed us to test the effects of salicylate within the same neurons, greatly enhancing our ability to also detect more subtle changes.^52^ We observed that salicylate reduced spontaneous activity in the IC and improved both the encoding of sinusoidally modulated (SAM) noise in the IC and its behavioral detection.

## Results

Using multielectrode recordings, we assessed the effects of salicylate on the spontaneous and sound-evoked activity of well-isolated single units in the IC of mice that were anesthetized with ketamine-xylazine. The same units were recorded both before and after injection of salicylate (250 mg/kg). At the end of the experiment, or shortly after the animal died, blood was collected to estimate the plasma concentration of salicylate (Figure S1A). The salicylate concentration was about 350 mg/L around the time most recordings were performed. Similar plasma concentrations have been observed previously in mice following injections of 200–400 mg/kg, but with much faster clearance.^53^

**Figure 1:**
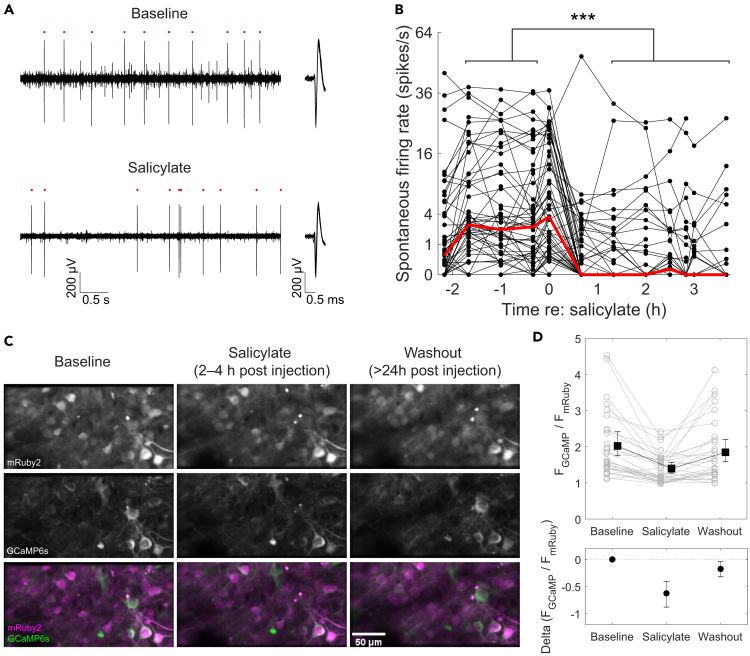
Salicylate suppressed spontaneous firing (A) Example unit activity at baseline (top panel) and salicylate (bottom panel). Red markers indicate the spikes belonging to the illustrated single unit. Left: 5 s trace from recording channel on which this unit had its largest amplitude. Right: Overlaid waveforms of spikes present in example trace. (B) Time course of salicylate effect on spontaneous firing rate (shown with square root scale) of the population of units sampled. Red trace shows the population median. Wilcoxon rank-sum test of the average spontaneous firing between baseline and salicylate revealed significant inhibition (∗∗∗ denotes *p* < 5.10^−8^). (C) Average fluorescence images of an example area during spontaneous activity in baseline (left), salicylate (center) and washout (right) periods. To facilitate the visual comparison, the max value in the GCaMP6s fluorescence images was scaled to be three times the max value in the corresponding mRuby2 fluorescence image. Scale bar in the lower right panel applies to all images. (D) Upper: fluorescence ratio (F_GcaMP_/F_mRuby_) in baseline, salicylate and washout conditions in the absence of auditory stimulation. Gray lines and symbols represent data from individual ROIs. Black lines and symbols represent mean, with error bars representing bootstrapped 95% confidence interval for the mean. Lower: mean differences of spontaneous fluorescence ratio compared to baseline condition and their bootstrapped 95% confidence intervals. See also Figures S1 and S2.

The spontaneous firing rate was determined from 8 to 12 recording periods of about 5 s of silence prior to the start of the first stimuli of a new recording set. Salicylate reduced (n = 6/55 units) or even completely abolished (n = 33/55) spontaneous firing (Figures 1A and 1B; median rate 2.5 to 0.0095 spk/s; Wilcoxon rank-sum test, *p* = 4.10^−8^). The other units showed brief inhibition and subsequent return to baseline firing (n = 2/55), increased spontaneous firing rate in the presence of salicylate (n = 7/55), or no change (*n* = 7/55). Histological reconstruction of the recording sites of electrodes stained with a fluorescent dye^49^ showed that the inhibition of spontaneous activity could be observed in the different subnuclei of the IC (*results not shown*).

To test whether the salicylate-induced reduction of spontaneous firing depended on anesthesia and to test whether this reduction was reversible, we also investigated spontaneous activity in awake animals using ratiometric *in vivo* two-photon imaging. In this case, neurons were identified across sessions based on their location and shape. Compared to baseline, the ratio between the activity-dependent GCaMP6s fluorescence and the activity-independent mRuby2 fluorescence was decreased 2–4 h after salicylate was injected in most cells (n = 30 ROIs, n = 2 mice; Figures 1C and 1D), and almost fully recovered >24 h after the last salicylate injection. In a previous study in which the effects of salicylate on spontaneous activity of IC neurons were studied, it was found that high-frequency units were exempted from inhibition.^24^ Figure S1B illustrates the relation between the relative change in spontaneous firing rate upon salicylate administration and the CF of the neuron at baseline. No significant relationship was found between the baseline CF of the neuron and the change in spontaneous firing rate (linear regression model, n = 47 units, *p* = 0.15). Six units were excluded from this analysis due to their lack of spontaneous firing in both baseline and salicylate recording periods. Even though the number of high-frequency units is limited, the effect of salicylate seems also to be present for high-frequency units. We conclude that salicylate reversibly reduced spontaneous activity of most IC neurons both in awake and anesthetized animals.

**Figure 2:**
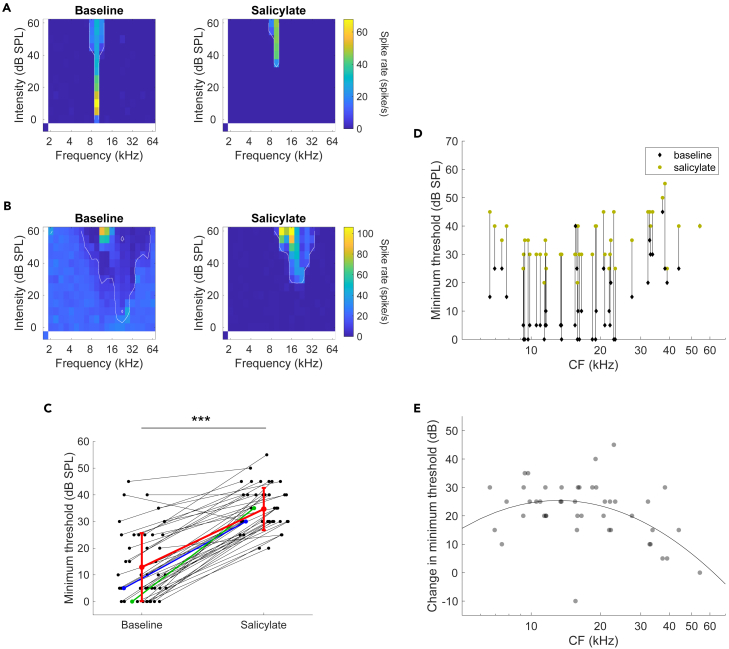
Salicylate increased the MT of IC neurons (A) A sharply tuned single unit with a CF of 9.5 kHz showed a clear elevation of the MT from 0 (left panel) to 35 (right) dB SPL in the presence of salicylate. (B) A more broadly tuned unit showed an elevation of MT from 5 (left) to 30 (right) dB SPL and a shift of CF from 22.6 to 19 kHz. White outlines in A and B indicate the borders of significant tuning obtained from the FACA. (C) Most units showed an increase in MT (paired two-tailed t-test, ∗∗∗ denotes *p* < 10^−4^). Blue and green dimensionality lines show the change in MT of example units shown in (A) and (B), respectively. Red line shows the mean MT in baseline or salicylate. Error bars denote SDs. (D) Changes in MT were observed across the range of CFs. Relation between threshold shifts induced by salicylate and CF under baseline conditions. CFs were slightly jittered for display to avoid overlap. (E) Relationship between the magnitude of the salicylate threshold shift and the unit’s baseline CF. A parabolic function was fitted to the data: $\Delta MT=-55.66{\left(\log\;CF\right)}^{2}+124.48\;\log\;CF-44.21$, where ΔMT is the change in MT in dB SPL and CF is expressed in kHz. The same jitter in CF from (D) was applied to each datapoint. Related to Figure 1.

We computed a pure-tone FRA for each unit. FRAs showed a great variety, in agreement with previous results.^49^^,^^50^ Examples of a sharply and a more broadly tuned neuron are illustrated in Figures 2A and 2B, respectively. A clear increase in MT from 12.9 ± 12.8 dB sound pressure level (SPL) to 34.7 ± 7.9 dB SPL was observed in the presence of salicylate. Figure 2C shows that in the neurons with an FRA that could be defined both before and in the presence of salicylate, this elevation of MT was observed in almost every (44/45) neuron (two-tailed paired t-test, *p* = 3.5 x 10^−18^). A small but significant decrease in CF was observed in the presence of salicylate (ΔCF = −0.23 ± 0.20 octave, two-tailed paired t-test, n = 45 units, *p* = 0.0215).

Additionally, we evaluated the relationship between the change in MT and the neuron’s CF. Changes in MT of individual neurons plotted against their baseline CF showed on average higher thresholds at the lower and upper edges of the sampled CFs (Figure 2D). We found that a parabolic curve fit to the relation between MT change and CF represented the trend well (Figure 2E).

**Figure 3:**
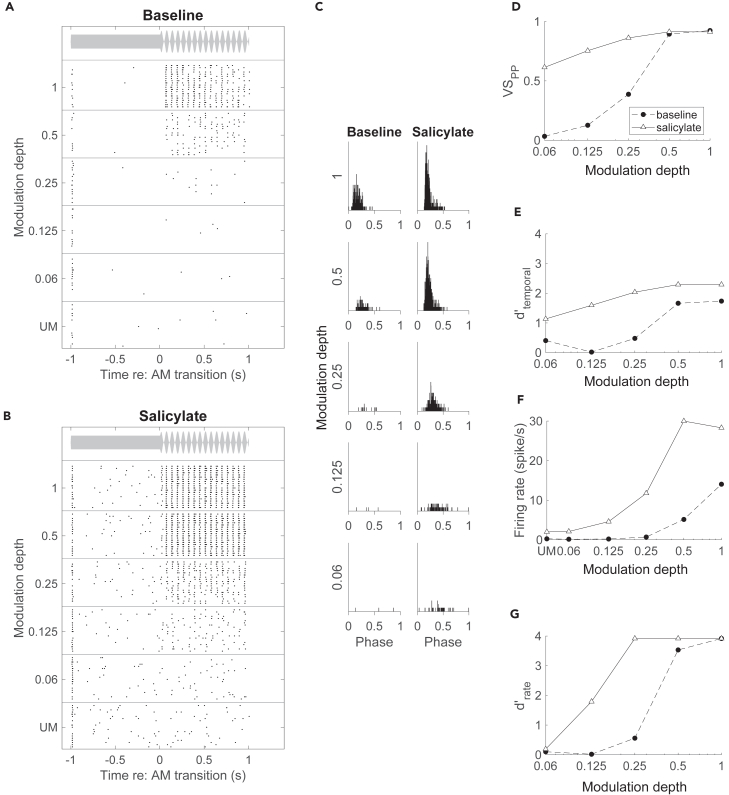
Salicylate improved phase locking to SAM noise at low modulation depths Dot raster plots of the response to 60 dB SPL, 16 Hz SAM noise in an IC single unit during baseline (A) and in the presence of salicylate (B), with the trials organized by increasing modulation depth. Trial onset, i.e., the start of the UM noise, was at −1 s. Stimulus envelope is shown at the top of the panels. Note the earlier response to the AM in the presence of salicylate. (C) Cycle histograms display phase-locking of spikes to the modulation period during the baseline period (left) and after salicylate (right). Full y axis scale is 0.12 spike/cycle/bin; bin size is 0.13 ms. The effect of salicylate on temporal coding of AM is shown by its effect on (D) VS_PP_, and (E) d’ calculated from VS_PP_, while its effect on rate coding is shown in (F) for the raw firing rate and in (G) for the calculated d’. See also Figure S2.

We tested the effect of salicylate on the responses to noise that was SAM at different frequencies and depths. Most units became more sensitive to AM. Figure 3 shows the effect of salicylate in one such unit at a modulation frequency of 16 Hz. Prior to salicylate, SAM noise triggered only few spikes at a modulation depth of 0.06 or 0.125, similar to the response to UM noise, but increasing spike rates were observed at higher modulation depths (Figures 3A–3G). In the presence of salicylate, AM-evoked firing already increased at a modulation depth of 0.125 (Figures 3B–3G). The VS_PP_ also showed a clear increase at low modulation depths, whereas at high modulation depths, VS_PP_ plateaued at values close to 1 (Figure 3D). This cell therefore showed a clearly enhanced response to AM, but it is not entirely clear to what extent its phase locking improved, or whether the increased VS_PP_ was merely a consequence of the increased firing rate.

To resolve this distinction, it is helpful to look at a unit with a high firing rate irrespective of modulation depth both before and in the presence of salicylate (Figure S2). The baseline firing rate of this unit was similar across modulation depths, with a small decrease at full depth (Figures S2A and S2F). Evidence for rate coding was obtained from receiver operating characteristic (ROC) analysis (Figure S2G). Salicylate increased the firing rate across all modulation depths (Figure S2F), resulting in a leftward shift of the sensitivity curve (Figure S2G). Also, in this unit phase-locking improved in the presence of salicylate (Figures S2A–S2C). VS_PP_ clearly increased after salicylate, except at full modulation depth (Figure S2D). The relatively low VS_PP_ can be explained by the two different preferred latencies shown in the cycle histogram (Figure S2C). Despite this, the temporal sensitivity curve sharply increased to a ceiling value for all modulation depths except 0.06 (Figure S2E).

**Figure 4:**
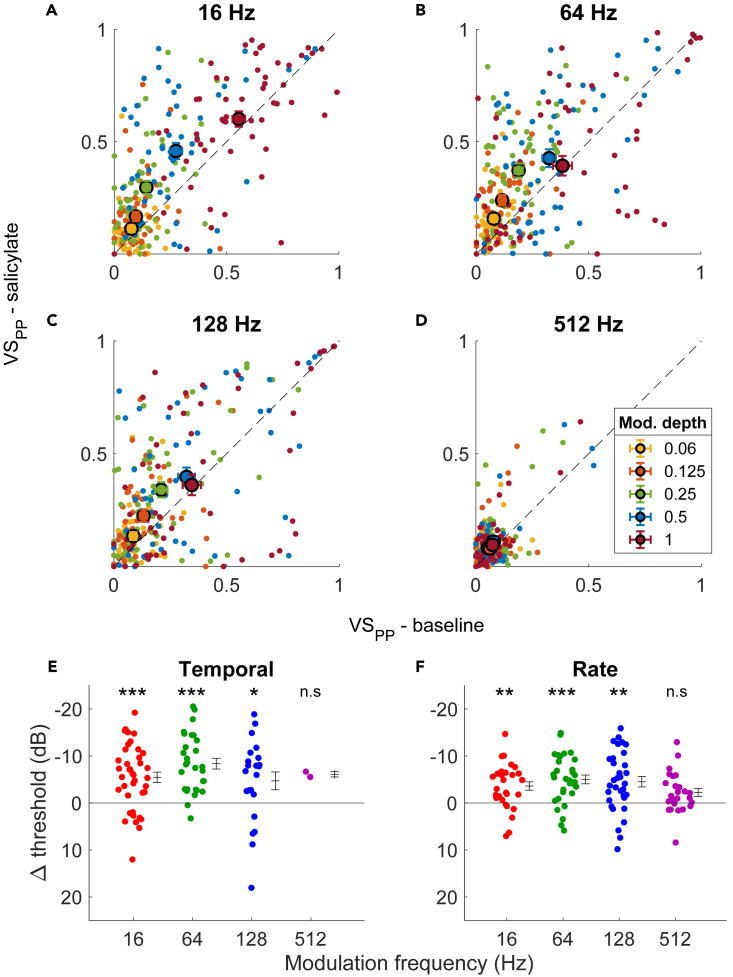
Population effect of sodium salicylate on AM encoding (A–D) Unit VS_PP_ values post-salicylate plotted against baseline at each modulation depth and modulation frequency for 60 dB SPL stimuli. Units showing detection of AM before and after salicylate on average showed a decrease in threshold of both (E) temporal and (F) rate encoding. ∗*p* < 0.05, ∗∗*p* < 0.01, ∗∗∗*p* < 0.001, n.s = not significant, paired t-test with multiple comparison correction. Values represent mean ± SEM. See also Figures S4 and S5.

We next evaluated the effects of salicylate on sound responses at the population level. Firing rates during UM noise were similar during control and in the presence of salicylate (*p* = 0.70, Wilcoxon signed rank test, n = 55). For SAM noise, at the lowest three modulation frequencies we observed an overall increase in VS_PP_ with salicylate—with the largest effect at the middle modulation depths and little or no change at the lowest (0.06) and the highest (1) modulation depth (Figures 4A–4C). At 512 Hz, while most units showed poor phase-locking both before and in the presence of salicylate, the units that did show a change generally tended to show better phase-locking with salicylate (Figure 4D). To illustrate the effect of salicylate on AM detection, VS_PP_ and AM-firing rate were converted to a sensitivity index (d’) for temporal and rate coding, respectively. The detection threshold was defined as the modulation depth where the (interpolated) sensitivity (d’) equaled 1. This detection threshold was lower in the presence of salicylate for both temporal (Figure 4E) and rate (Figure 4F) encoding of AM in most units in which the detection threshold could be calculated both before and in the presence of salicylate. These decreases were significant at 16, 64, and 128 Hz for both temporal (16 Hz: Δthr = −5.46 ± 1.11 dB, *p* = 0.00012; 64 Hz: Δthr = −8.37 ± 1.15 dB, *p* = 0.00002; 128 Hz: Δthr = −4.71 ± 1.89 dB, *p* = 0.020, 512 Hz: Δthr = −6.10 ± 0.56 dB, *p* = 0.24301; paired t-test corrected for multiple comparisons) and rate thresholds (16 Hz: Δthr = −3.62 ± 0.89 dB, *p* = 0.00012; 64 Hz: Δthr = −5.02 ± 0.93 dB, *p* = 0.00002; 128 Hz: Δthr = −4.52 ± 1.11 dB, *p* = 0.020, 512 Hz: Δthr = −2.24 ± 0.86 dB, *p* = 0.12568; paired t-test corrected for multiple comparisons). Thresholds could not be calculated for all units (Figure S3A). At most modulation frequencies there were more units that had a detection threshold in the presence of salicylate but not before than the other way around. This was true for both temporal (Figure S3A) and rate encoding of AM (Figure S3B), suggesting that the improvement of AM encoding was not due to a bias resulting from an inability to calculate a threshold in some units. Improved AM encoding could also be clearly observed in multiunit activity within the IC (*results not shown*).

**Figure 5:**
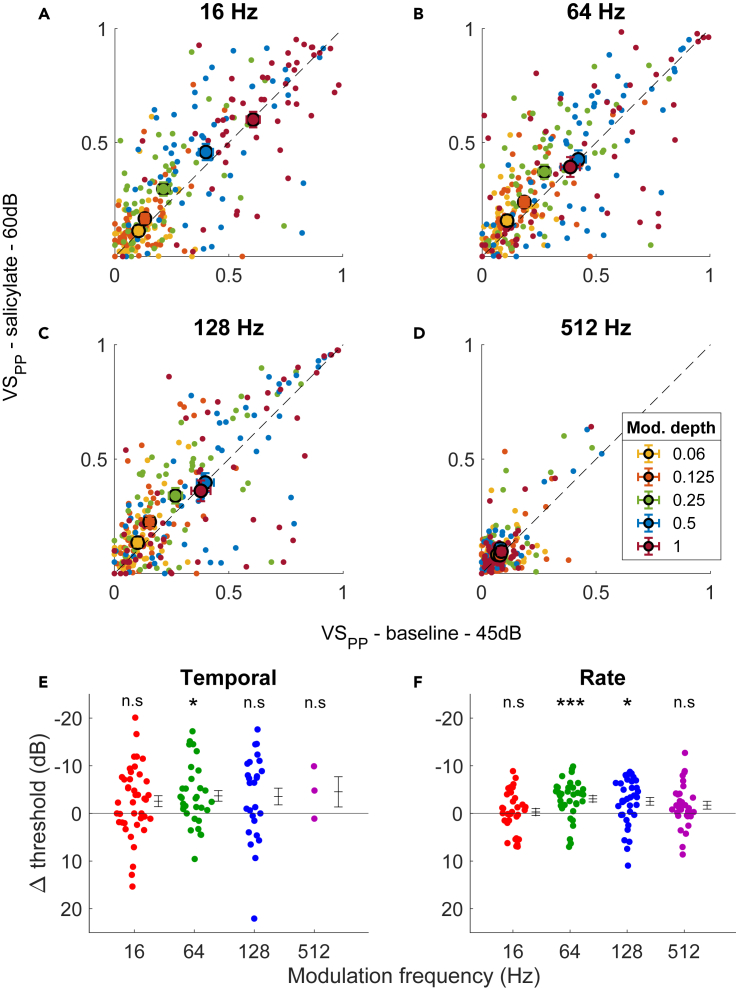
After compensating for the higher hearing threshold, salicylate still lowers the AM detection threshold (A–D) Comparison of VS_PP_ for 60 dB SPL SAM noise in the presence of salicylate with VS_PP_ evoked by 45 dB SPL SAM noise under baseline conditions. Small circles denote individual cells, large symbols averages ±SEM. (E and F) Differences in thresholds between 45 dB control condition and 60 dB salicylate for temporal (E) and rate (F) coding. ∗*p* < 0.05, ∗∗*p* < 0.01, ∗∗∗*p* < 0.001, ns = not significant, paired t-test with multiple comparison correction. Error bars show mean ± SEM. Related to Figure 4.

What is the mechanism behind the improvement in AM detection in the presence of salicylate? Salicylate’s effect on the MT of the units (Figure 2) raises the possibility that the effect of salicylate on AM detection was secondary to its induction of hearing loss. Specifically, it might be that in the presence of salicylate a larger fraction of the modulation depths would now straddle the hearing threshold. We will call this alternative explanation the iceberg effect. To assess this possibility, we repeated our analysis described previously, except we now compared the 60 dB SPL post-salicylate injection values with the 45 dB SPL baseline values. This intensity increase was similar to the average increase in MT of the single units (Figure 2). While not as strongly deviating from the unity line as the matched-intensity comparison, the VS_PP_ was still higher in the presence of salicylate (Figures 5A–5D). The change in thresholds also resembled the matched-intensity analysis for both the temporal- and rate-encoded thresholds (Figures 5E and 5F). These decreases in thresholds in temporal encoding were significant only at 64 Hz (16 Hz: Δthr = −2.57 ± 1.15 dB, *p* = 0.41; 64 Hz: Δthr = −3.68 ± 1.12 dB, *p* = 0.019; 128 Hz: Δthr = −3.55 ± 1.75 dB, *p* = 0.37, 512 Hz: Δthr = −4.53 ± 3.17 dB, *p* = 0.32; paired t-test, *p*-values Bonferroni-corrected for multiple comparisons). The threshold decreases were significant for rate encoding for modulation frequencies 64 and 128 Hz (16 Hz: Δthr = −0.29 ± 0.73 dB, *p* = 1.0; 64 Hz: Δthr = −3.06 ± 0.66 dB, *p* = 0.00039; 128 Hz: Δthr = −2.53 ± 0.81 dB, *p* = 0.029, 512 Hz: Δthr = −1.72 ± 0.81 dB, *p* = 0.34; paired t-test, *p* values Bonferroni-corrected for multiple comparisons). Moreover, there were more units with detectable thresholds in the presence of salicylate than under baseline conditions, except for the temporal threshold at 512 Hz (Figures S3C and S3D).

**Figure 6:**
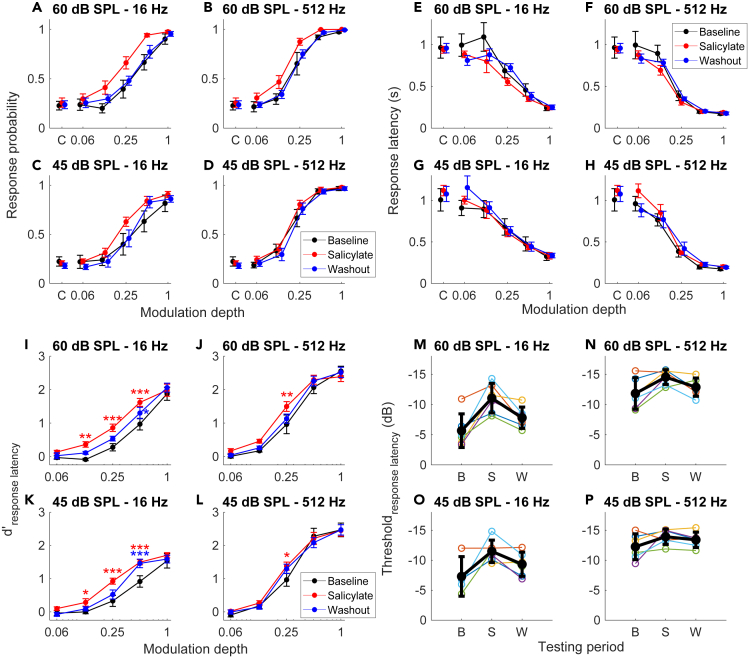
Improved behavioral AM detection in the presence of salicylate (A–D) Relation between response probability and modulation depth at two sound intensities and two modulation frequencies during baseline, in the presence of salicylate and after its clearance. The false alarm rate obtained from catch trials is indicated by “C” on the x axis. (E–H) As A–D, but showing the relation between average median response latencies and SAM noise modulation depth. Values represent mean ± SEM. (I–L) Relation between AM behavioral sensitivity (d’) as calculated from ROC analysis of the response latencies and modulation depth. ∗*p* < 0.05, ∗∗*p* < 0.01, ∗∗∗*p* < 0.001, for the interaction between condition (salicylate or washout) and modulation depth, compared against baseline using a linear mixed-effect model for each panel. (M–P) Thresholds calculated from d’. “B” is baseline, “S” is salicylate, “W” is washout. Values represent mean ± SEM.

To test whether the improved neural encoding of the AM stimuli also led to improved detection of these stimuli by the animals, we trained mice to respond with a lick to the transition from UM to SAM noise. Correct detections were rewarded, incorrect licks were punished with a timeout.^49^ We only tested the modulation frequencies 16 and 512 Hz to ensure that a sufficient number of trials were obtained during salicylate sessions. The performance of the mice was quantified by calculating their hit rate (HR) and their false-alarm rate (FA; the response probability for the catch trials). The relation between response probability and modulation depth followed a sigmoidal curve (Figures 6A–6D). No significant difference in FA was found across stimulus intensity or condition (i.e., baseline, salicylate or washout) based on a two-way ANOVA (n = 6 mice; for intensity: *p* = 0.24; for condition: *p* = 0.64; for intensity∗condition: *p* = 0.91). At full modulation depth, HR was close to 1, whereas at a modulation depth of 0.06, it was close to FA. At 60 dB SPL, a clear increase in HR was seen for the middle modulation depths in the presence of salicylate, whereas little or no change was seen for the highest modulation depths, where HR was already close to 1 under control conditions, or at a modulation depth of 0.06 (Figures 6A and 6B). These effects are thus reminiscent of the effects of salicylate on AM encoding by IC units (Figure 4). For 45 dB SPL stimuli, a change also seemed to be present at 16 Hz (Figure 6C), but not at 512 Hz (Figure 6D).

The response latency provided additional information about the AM detection by the mice. The animals typically showed an inverse relation between modulation depth and the average median response latency (Figures 6E–6H). For the 60 dB SPL stimuli, response latency at the middle modulation depths appeared to improve in the presence of salicylate.

Upon conversion of the response latency to d’ by ROC analysis, AM behavioral detection showed an improvement after salicylate compared to baseline at the modulation frequency of 16 Hz (Figures 6I and 6K). These improvements were mainly observed at the middle modulation depths. This effect was present at both high and low sound intensities. The improvement was also partially reversed following the washout period. At the modulation frequency of 512 Hz, this salicylate-induced improvement in AM detection was also observed, but the return to baseline sensitivity was less apparent at the lower sound intensity (Figures 6J and 6L).

To quantify the change in behavioral AM detection, we defined a detection threshold as the lowest modulation depth at which d’ equaled 1. By assessing the change in thresholds across the testing periods, we found that salicylate overall lowered the thresholds (−5.0 ± 0.9 dB; *p* < 0.001, linear mixed-effect model with linear hypothesis testing) compared to baseline measurements (Figures 6M–6P). The effect remained significant when examined at both the 16 Hz (−5.9 ± 0.9 dB; *p* < 0.001; Figures 6M and 6O) and 512 Hz (−4.3 ± 1.6 dB; *p* = 0.049; Figures 6N and 6P), with the intensities pooled. Thresholds obtained after washout showed no difference with baseline measures (−1.7 ± 0.9 dB; *p* = 0.34).

To assess the iceberg effect explanation for the salicylate effect, the behavioral threshold differences were compared between baseline and salicylate at intensities of 45 and 60 dB SPL, respectively. At 16 Hz, thresholds were significantly lower (−5.9 ± 1.3 dB, *p* < 0.001). At 512 Hz, the trend of a reduced threshold was still present, but did not reach statistical significance (−2.6 ± 1.3 dB, *p* = 0.23).

Reduced inhibition in the auditory system has been suggested to be an important mechanism underlying the central effects of salicylate on neural excitability.^4^ To investigate whether salicylate decreases inhibitory inputs to IC neurons, the FRAs were not very informative because of the strong reduction in spontaneous activity (Figure 2B). We therefore estimated the STRF of IC neurons using DRCs as a stimulus; DRCs allow to look at the interaction between tones.^38^^,^^39^^,^^40^ We presented DRC stimuli both before and after injection of salicylate. Forty-three of the 55 well-isolated single units (from 8 animals) could be identified during both baseline and salicylate DRC recordings. Of these, four had insufficient signal power in at least one of the recordings.^54^ Figures S4A and S4B shows a typical DRC stimulus and Figures S4C and S4D the response of two example units to it in the same animal under baseline condition. An STRF fitted to the data from these two units reveals their frequency tuning and response latencies (Figures S4E and S4H).

**Figure 7:**
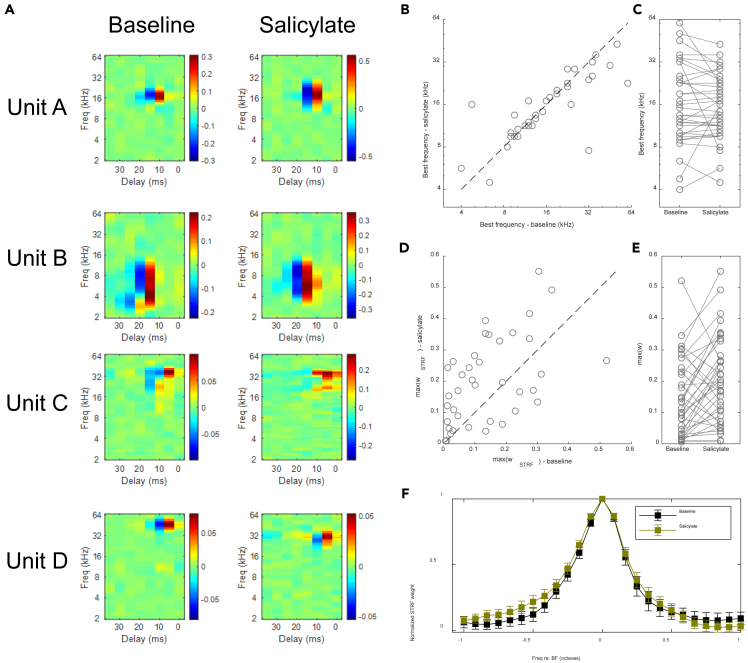
The STRFs of IC neurons remained stable in the presence of salicylate (A) Example STRFs of 4 example neurons before (left) and after (right) the injection of salicylate. Units A and B correspond to the same units in Figure S4. The STRF of the first three examples remained largely similar, with correlation coefficients 0.83, 0.78 and 0.45, respectively. The last example had a shift in BF, and the correlation coefficient between STRFs was only 0.04. (B and C) The best frequencies (BF) of IC neurons remained similar in the presence of salicylate. (B) Scatterplot of BFs in salicylate condition against baseline condition. Broken line is the identity line. (C) Change in BFs upon salicylate injection. (D) Scatterplot of maximum STRF weight (max(w_STRF_)) in salicylate condition against baseline condition. Broken line is the identity line. (E) Change in max(w_STRF_) upon salicylate injection. (F) Averaged BF-aligned STRF weights before (black) and after (brown) salicylate injection. Error bars represent SEM. Related to Figures 5 and 6.

STRFs remained largely similar following salicylate injection (*r* = 0.60 ± 0.20). Figure 7A shows four example units ranging from the best correlated (units A, B) to the worst case (unit D). Best frequencies (BF) also remained similar (Figures 7B and 7C; change in BF −0.07 ± 0.55 octaves; one-sample t-test: *p* = 0.43). There was, however, an overall increase in the maximum weight of the STRF (Figures 7D and 7E; baseline: 0.14 ± 0.12 vs. salicylate: 0.20 ± 0.14; paired t-test: *p* = 0.006), which will be further discussed in the following section. The tuning broadness was also similar between the two conditions (Figure 7F).

**Figure 8:**
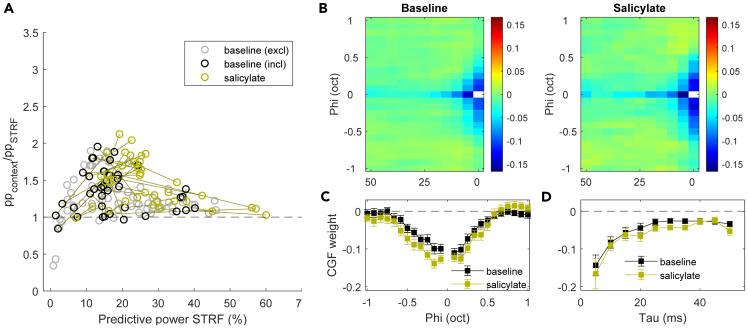
Context STRF model suggests limited increase in sideband inhibition and adaptation (A) Ratio of predictive powers of the context model vs. simple STRF model (pp_context_/pp_STRF_) are in general above one, indicating that the inclusion of the context gain field (CGF) provided a better fit to the data for both baseline (black and gray symbols) and salicylate (gold symbols) conditions. Individual lines connect recordings from the same unit. (B) Average CGFs for baseline (left) and salicylate (right) conditions are qualitatively similar, with negative weights along the 0-tau and 0-phi bins. (C) Average CGF weights for baseline (black) and salicylate (gold) conditions along the 0-tau bins. Error bars indicate SEM. (D) Same as C but for 0-phi bins. See also Figures 7 and S4.

To investigate the interactions between simultaneously presented tones, we fitted the data to a quadratic STRF model where the local spectral “context” was taken into account.^41^ Hereafter, we will refer to this model as the “context model” and the basic STRF model simply as the “STRF model”. The context model had better predictive power than the STRF model (Figure 8A), and produced principal receptive fields (PRF) which were highly similar to the corresponding STRFs (Figures S4F and S4I; *r* = 0.95 ± 0.03; range 0.80–1.00). The context model additionally provided a context gain field (CGF) that reveals how the presence of additional tones in the spectrotemporal vicinity affected a neuron’s tone response (Figures S4G and S4J). The average CGF of IC neurons showed a negative band along the zero-frequency offset (φ) and the zero temporal offset (τ) line. The former reflects adaptation to long tones and the latter represents inhibition by nearby tones. Both remained qualitatively similar after salicylate injection with slightly stronger lateral inhibition (Figure 8C) and longer adaptation (Figure 8D).

**Figure 9:**
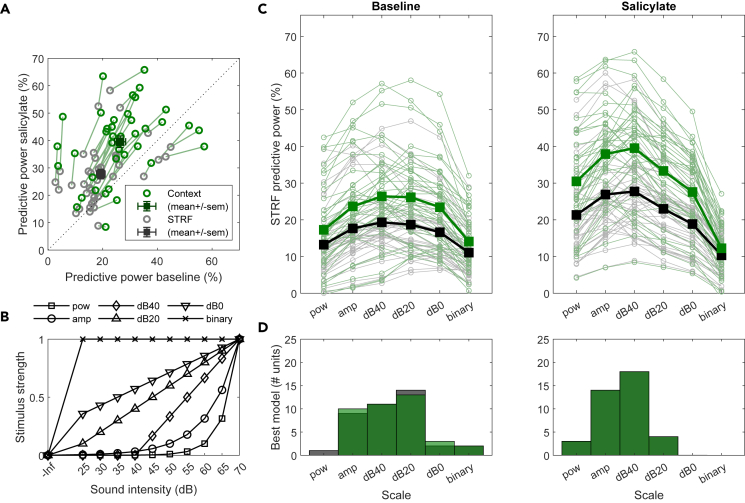
Improvement in predictive power in the presence of salicylate suggests a linearization of the response to sound (A) Normalized predictive power of both simple (gray) and context (green) STRF models improved in salicylate condition compared to baseline. Solid line is the identity line. (B) Different input scalings used for obtaining STRFs. (C) Predictive power of simple (gray/black) and context (green) STRF models under different input scaling. (D) Histograms of input scale that provided the highest predictive power for each unit with the simple (gray/black) or context (green) STRF model. More linear scales were favored in salicylate condition. See also Figures 7 and S4.

One interesting observation is that both the STRF and the context model had higher predictive power in the presence of salicylate (Figure 9A). We postulate that the responses of IC neurons became more linear, as STRF models assume a linear increase in response as a function of stimulus strength. We further tested this by repeating the fitting using a variety of intensity scales representing different degrees of linearity (Figure 9B). The responses of most IC neurons were best predicted by either the standard amplitude scaling (“amp”) or a decibel scaling starting at 40 dB SPL (“dB40”) (Figures 9C and 9D). More importantly, the best input scaling shifted toward more linear regimes in the presence of salicylate: the number of neurons that were best fit with a linear model (“pow” or “amp”) increased, while the number of neurons that were best fit with more compressive models (“dB0”, “dB20”) decreased (Figure 9D). On average, the amp and dB40 scales became the best input scaling in terms of average predictive power (Figure 9C) and the number of units that were best predicted by this scaling (Figure 9D).

**Figure 10:**
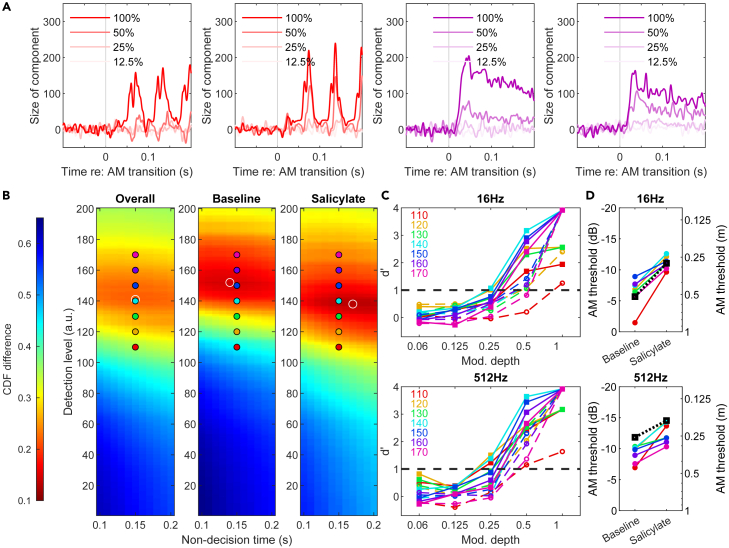
Improved behavioral AM detection in the presence of salicylate by a threshold-crossing model based on IC population responses (A) Evolution of trial-averaged population activities projected onto the principal AM-encoding component in response to various AM stimuli in baseline (left) and salicylate (right) conditions. (B) Goodness-of-fit heatmaps for choices of detection levels and non-decision time in the simulation. Color indicates RMS difference between CDFs of simulated and behavioral response latencies. Baseline and salicylate conditions were evaluated together (left) or separately (middle: baseline; right: salicylate). White circles indicate parameter sets with the smallest CDF difference. Color dots represent parameters used for data in C. (C) d’ derived from simulated response latencies for 16 Hz (upper) and 512 Hz (lower) in baseline (dashed lines) and salicylate (solid lines) conditions. Different colors correspond to different detection levels indicated in the legend. (D) AM detection threshold derived from d’ = 1 for 16 Hz (upper) and 512 Hz (lower) at various detection levels (colored lines as in C), in comparison with the behavioral threshold (black dashed lines).

The improvement in AM behavioral detection was also reflected in the population coding of IC neurons. Using a simple dimensionality reduction model,^49^ we investigated how the AM-encoding component of the population activity evolves in time as a function of modulation depth, before and in the presence of salicylate (Figure 10A). The population activities (n = 55 neurons) for both salicylate and baseline conditions were projected onto the AM-encoding component for the baseline condition. For both low (16 Hz) and high (512 Hz) modulation frequencies, the trial-averaged AM component showed a marked increase in amplitude in response to intermediate modulation depths (e.g., 0.5, 0.25), suggesting an improvement in overall sensitivity.

Next, we simulated the trial-by-trial detection of SAM noise with a simple threshold-crossing method.^49^ While the precise response times in baseline and salicylate conditions were better fitted with different detection levels (Figure 10B), conversion to the signal detection measure d’ (Figure 10C upper) and subsequently AM detection threshold robustly recapitulated its improvement in the presence of salicylate (Figure 10C lower). This result was consistent across a large range of values for the detection level parameter (different colored dots and lines in Figures 10B and 10C). With that, we conclude that the improved AM detection in the presence of salicylate is well reflected in the population response in the IC.

## Discussion

We tested the effects of systemic salicylate on the spontaneous and sound-evoked single unit activity in the mouse IC. By recording from the same, well-isolated single units both in the absence and the presence of salicylate, we were able to detect even small changes in their properties. Both in awake and in anesthetized animals, salicylate induced a large decrease in spontaneous activity. Salicylate also induced about a 20 dB SPL increase in the MT of IC units. Both an increase in the vector strength of phase-locking and improved rate coding were seen in the presence of salicylate, especially at low modulation depths. The improved encoding of the AM stimuli by the IC neurons was also reflected in a more sensitive behavioral detection of AM stimuli. Experiments in which we presented random combinations of brief tones at different intensities suggested that the main mechanism underlying the improved AM detection was a decrease in the compressive nonlinearity of the cochlear responses, presumably due to salicylate’s effect on outer hair cells. This cochlear effect is expected to result in a more linear relationship between sound intensity and cochlear output, leading to larger contrasts during AM stimuli. This phenomenon is expected to be important when studying the effects of salicylate at different sound intensities, and is therefore important to take into account when studying hyperacusis.

### Was the salicylate dose sufficiently high to induce tinnitus in our experiments?

We observed a clear decrease instead of an increase in spontaneous firing, as discussed further in the following section. This raises the question whether the salicylate dose was sufficiently high for us to reasonably assume that the mice had tinnitus in our experiments. While only behavioral tests can definitively answer this question, based on the literature the salicylate dose we used in our experiments should in principle be sufficiently high to induce tinnitus in mice. In humans, salicylate can induce tinnitus already at a lower concentration than for inducing hearing loss.^1^ In normal hearing humans, tinnitus can be reliably observed at salicylate plasma concentrations as low as 100 mg/L.^2^ At this concentration, hearing loss was typically only about 10 dB,^2^ less than what was observed in our study. In earlier work in mice using doses somewhat higher (300–350 mg/kg) than what we used here, behavioral evidence for the presence of tinnitus in an active avoidance task was reported.^55^ In addition, at salicylate concentrations similar or higher to what we employed, evidence for the presence of tinnitus in mice has been obtained using the gap-prepulse inhibition of the acoustic startle reflex test.^28^^,^^35^^,^^56^^,^^57^^,^^58^ Even though its suitability as a tinnitus test is debated (reviewed in Galazyuk et al.^59^) this test is probably the most frequently employed behavioral tinnitus test. We therefore conclude that based on the literature in both humans and mice, it can be argued that the salicylate dose should have been sufficiently large to induce tinnitus in our present experiments.

### Effect of salicylate on spontaneous activity

An increase in the spontaneous firing rate at the level of the IC has been viewed as an electrophysiological proxy for tinnitus.^60^ It was therefore surprising that the spontaneous activity of most IC neurons showed a clear decrease in the presence of salicylate. In most IC neurons, spontaneous action potentials are triggered by spontaneous EPSPs,^61^ suggesting that the reduction of spontaneous firing is likely to be due to a decrease in the frequency or amplitude of spontaneous EPSPs.

In previous studies, the effects of systemic administration of salicylate on spontaneous activity within the IC were variable. A reduction in spontaneous activity was also observed in previous studies from the mouse CNIC.^24^^,^^28^ Interestingly, Ma et al.^24^ found no clear effects on high-frequency units (>27 kHz) (see in the study by Muller M et al. and Eggermont et al.^25^^,^^62^), which was not apparent in our data (Figures 2D and 2E) or those of ref.^28^. In contrast, salicylate induces a large increase in the spontaneous activity of most units in the IC of both the anesthetized and awake guinea pig^19^^,^^20^^,^^21^ and the external nucleus of the rat IC.^18^ Studies using metabolic markers also obtained evidence for increased spontaneous activity in the IC in awake rats,^63^^,^^64^ but a decrease in the gerbil IC.^65^

The opposite effects of salicylate on spontaneous firing in the IC are unlikely to be due to an interaction with anaesthetics, since we observed the inhibitory effects both in awake and in anesthetized animals, and the same held true for the stimulatory effects in guinea pigs and rats.^18^^,^^19^^,^^20^^,^^21^^,^^63^^,^^64^ It was also unlikely to be due to a different salicylate dosage, since results were consistent in the rats or guinea pigs at both higher and lower dosages than the dosages we or others^24^^,^^28^ employed in the mouse.^18^^,^^19^^,^^21^^,^^63^^,^^64^^,^^66^ Also, the plasma concentration of salicylate of about 350 mg/L in our experiments was lower than in the rat study,^18^ but similar as in the earlier studies in the guinea pig, in which a clear increase in spontaneous activity was observed.^20^^,^^21^

Differences in the IC subregion that was recorded also seems an unlikely factor, since increases in spontaneous activity were observed throughout the IC in rats and guinea pigs,^18^^,^^19^^,^^20^^,^^21^^,^^63^^,^^64^ whereas we also saw decreased spontaneous activity throughout large parts of the IC, including the dorsal IC.

What does the observed decrease in spontaneous frequency in the IC mean for the presence of tinnitus in our study? It is possible that spontaneous frequency increased in other regions of the auditory system. Alternatively, increased spontaneous activity in the auditory system is not a good biomarker for tinnitus. Some authors have noticed a discrepancy between the presence or timing of an increase in spontaneous activity and the behavioral evidence for the presence of tinnitus.^67^^,^^68^^,^^69^ Alternatively, changes in the firing pattern or firing synchrony^17^^,^^18^^,^^23^ may be more important than an increased firing frequency. However, apart from the decrease in spontaneous firing, we did not observe any obvious changes in firing patterns such as increased burst firing in our dataset. Another possibility is that salicylate did not induce tinnitus in our experiments, as the opposing effects of salicylate on spontaneous activity in the IC in different species do suggest that species differences in the ability of salicylate to induce tinnitus are a possibility.

Interestingly, in the guinea pig IC, local administration of salicylate in the IC resulted in a sustained decrease in spontaneous activity, instead of the increase observed following systemic administration.^19^ This suggests that in the guinea pig or rat, this local inhibitory effect is overcome by upstream changes, for example within the cochlear nucleus^23^ or at the level of the auditory nerve.^17^ More studies of the effect of salicylate in regions upstream and downstream of the IC would be needed to further delineate the cause of these opposing effects of systemic salicylate on spontaneous activity in the IC in different species. This could also provide more certainty about the suitability of mice (and possibly gerbils) for studying the mechanisms underlying salicylate-induced tinnitus.

### Effects of salicylate on AM sensitivity

We observed a clear improvement of both rate and temporal AM encoding in the IC. This was a robust effect, which was observed in most units. Apart from a study in the AC of awake cats showing improved phase locking to click trains,^26^ to our knowledge the effect of salicylate on neural AM encoding had not previously been studied in any detail.

A further argument in favor of its importance and robustness, was that the behavioral threshold for detecting AM also improved. The behavioral effect was similar to the electrophysiological effect, as it was most apparent at the intermediate modulation depths. We tested only two modulation frequencies and intermingled the trials with both frequencies to limit the number of days needed to complete the testing, but since the improvement was observed at both the low (16 Hz) and the high frequency (512 Hz), it seems likely to be a general effect. We believe that it is unlikely that the improved AM detection was due to increased motivation due to increased thirst caused by metabolic effects of salicylate,^70^ since the false alarm rate did not increase in the presence of salicylate. Whereas we cannot exclude that the effect of salicylate was nonauditory, the lack of a change in the false alarm rate suggests that the results were not due to changes in the response bias of the animal.

We will discuss three different potential mechanisms underlying the improved AM encoding: an iceberg effect due to the change in MT, a change in the balance between excitation and inhibition, and the loss of cochlear compression. Of these, we believe that the loss of cochlear compression is most pertinent.

### Iceberg effect

The iceberg effect entails that if the different intensities during an AM stimulus are straddling the MT, the AM encoding may be greatly enhanced. This seems less likely, since the modulation depths that were most relevant for the salicylate effect involved only a few dB of sound intensity differences, making it less likely to be influenced by a change in MT, which would be more of an all-or-none effect. Moreover, when we corrected for the hearing loss by comparing the detection in the presence of salicylate at 15 dB SPL higher intensity than before salicylate, the increased AM encoding was reduced, but could still be observed both electrophysiologically and behaviorally.

### Change in the balance between excitation and inhibition

A change in the balance between excitation and inhibition is considered to be an important mechanism underlying the central effects of salicylate on neural excitability.^4^ In our experiments, changes in side-band inhibition were difficult to assess in the presence of salicylate due to the strong decrease in spontaneous activity. The increase in MT also hampered the comparison of the shape of the FRA in the presence and absence of salicylate. We therefore used DRCs in combination with a local context model to investigate possible changes in inhibition.^41^ This model captures the effect of tones presented in close proximity in frequencies and time in the CGF. Analysis of the tone interaction suggested slightly more, instead of less, inhibition in the presence of salicylate, as shown by the more negative values in the CGF. One potential limitation was that we did not adjust the tone density and intensity range of the stimulus to control for the effect of a threshold shift on bandwidth estimation. However, bandwidth estimation was shown to be level tolerant with broadband stimuli,^71^ suggesting that the impact of the threshold shift should have been limited.

### Loss of cochlear compression

Even though we cannot exclude that the improved AM encoding is due to a central effect of salicylate, we favor it being a cochlear effect. Salicylate abolishes electromotility of outer hair cells,^9^^,^^10^^,^^11^ which linearizes the relation between amplitudes and sound level at the level of the basilar membrane.^6^^,^^7^^,^^8^ To what extent this loss of compressive nonlinearity at the level of the basilar membrane translates to central effects is still uncertain, as compressive nonlinearity was still observed at the level of the organ of Corti.^6^ The first positive peak of the ABR (P1) reflects the activity of the auditory nerve. The growth of P1 with sound intensity is similar in the absence and the presence of salicylate in mice,^56^ but this cannot be seen as a strong argument against linearization, since the relation between the growth of P1 and the presence or absence of compression is not straightforward.^72^^,^^73^ In our view, both the improved AM detection and the DRC results pointed in the direction of a linearization of the input-output relation at the cochlear level, which extended to the IC. We found that the prediction of neuronal responses to the DRC stimuli by the STRF, quantified as the normalized predictive power,^74^ improved in the presence of salicylate while using the same fitting parameters (Figures 9A and 9B). Moreover, response prediction improved upon using intensity scales that resemble sound pressure level changes (pow, amp) instead of a compressive regime such as the decibel scale (dB0, dB20).

The improved response prediction by the STRF suggests an increased linearity in the presence of salicylate, but the concomitant change in MT means that other explanations again also need to be considered. The input non-linearity has been investigated for AC neurons, showing that diverse shapes are needed to best describe the intensity scaling of different neurons,^75^ similar to the range of scales we found (Figure 9). To minimize the number of parameters, we used a few intensity scales with fixed, predefined weights for the different intensities for all neurons. All intensity scales we employed were monotonic, which may fit poorly to neurons with non-monotonic rate-level functions. In the presence of salicylate-induced threshold shifts, this non-monotonicity may be reduced, which might contribute to the improvement in the fits. Additionally, the rate-level function of auditory neurons adapts to the contrast presented in a DRC stimulus, which thus affects their response gain.^76^^,^^77^ This could be a potential confounder given our fixed intensity range of the DRC stimulus (25–70 dB SPL), which may translate to an effectively smaller contrast following a threshold shift in the presence of salicylate. Nevertheless, whereas we cannot exclude a contribution from the change in threshold to the improved STRF representation in the presence of salicylate, we believe that the linearization is the most important contributor, and that this also forms an explanation for the improved AM encoding.

Remarkably, despite the large body of data related to the auditory effects of salicylate,^1^^,^^4^ the loss of compressive nonlinearity has not received much attention in electrophysiological measurements in animals. Evidence was obtained that salicylate increases central gain, and this increase may be due to a reduction of synaptic inhibition.^14^^,^^15^^,^^32^^,^^78^^,^^79^^,^^80^^,^^81^ In some cases, the effect of salicylate on perceived loudness in rats is compatible with loudness recruitment.^82^ Decreased reaction times in an operant conditioning task with sounds of different loudness have been observed in the presence of salicylate.^82^^,^^83^^,^^84^^,^^85^ In these cases, the decreased reaction time was viewed to be a sign for increased loudness.

Interestingly, evidence for a linearization of the relation between sound input and responses in the presence of salicylate has also been obtained in humans. Both a linearization of the relationship between otoacoustic emissions and sound amplitude,^86^^,^^87^ and an increased sensitivity to small sound intensity increments^88^ were observed at high salicylate doses. These effects are thus in line with our present results in mice.

### Hearing loss and related disorders

The effects of salicylate and of sound overexposure on the electrophysiological properties of the IC bear some similarities. Both typically induce hearing loss, as evidenced by an increase of the MT of single IC units. The outer hair cells are not only the most important target of salicylate within the cochlea,^9^^,^^10^^,^^11^ but they are also easily damaged by sound overexposure.^89^^,^^90^ Owing to their unique electromotility properties, outer hair cells have an important role in the compressive nonlinearity of the cochlea, and a loss of outer hair cells will reduce cochlear gain at lower intensities, leading to a steeper than normal growth of loudness;^91^ this has been named loudness recruitment.^92^ Also in humans, similarities between “ordinary” sensorineural hearing loss and hearing loss due to salicylate toxicity have been observed.^93^ Interestingly, several lines of evidence indicate that changes in AM detection accompany sound-induced hearing loss as well. Increased sensitivity to envelope modulations can often be observed in hearing-impaired listeners.^94^ Using electrophysiology, enhanced neural synchronization to envelope modulations has been detected in hearing-impaired subjects at multiple stages of the central auditory system.^95^^,^^96^^,^^97^ Speech perception performance is generally inversely related to increased sensitivity to envelope modulations.^95^^,^^98^^,^^99^^,^^100^ The increased envelope encoding may lead to an imbalance in the envelope-to-fine structure representation in hearing-impaired listeners.^96^

Increased neural synchronization to envelope modulations was found in auditory nerve fibers of chinchillas following sound overexposure.^101^ Immediately following acute hearing loss, sound envelope coding was also enhanced in low-CF units in the guinea pig IC.^102^ Moreover, envelope coding of auditory evoked potentials recorded from the scalp surface was also enhanced following noise-induced hearing loss in chinchillas.^103^ Finally, neonatal noise exposure results in higher phase locking to SAM noise in the rat AC.^104^ We conclude that the salicylate-induced effects on AM encoding resemble what has been observed following sound overexposure, and that loudness recruitment due to outer hair cell (OHC) malfunction is a likely underlying mechanism. However, salicylate has also been shown to increase central gain.^12^^,^^83^ An increase in central gain may therefore have contributed to the enhanced responses to SAM noise, and measurements along the peripheral auditory system would be needed to quantify the relative contribution of both putative mechanisms. Additionally, local application of salicylate^13^^,^^15^^,^^19^ could help to further pinpoint the location of the increased encoding of SAM noise.

### Functional consequences of improved envelope coding

If, as argued previously, the improved neuronal and behavioral envelope coding is due to the abolishment of cochlear compression, this will severely reduce the dynamic range of the auditory system. This can be expected to have deleterious consequences, as exemplified by the decreased ability to detect speech in noise in humans with magnified envelope coding due to cochlear deficits.^95^^,^^98^^,^^99^^,^^100^ We therefore suggest that the consequences of hearing loss for envelope coding in the central auditory system will depend on which structures are most affected. With OHC deficits, one expects SAM detection to become better, whereas with auditory neuropathy, it would become worse.

### Relation with tinnitus and hyperacusis

The improvement of envelope coding does not have an obvious link to tinnitus, since tinnitus subjects typically show worse AM detection than controls.^105^ We conclude that the improved AM encoding may instead be related to loudness recruitment, but loudness recruitment is again not necessarily associated with tinnitus.^106^ Whereas loudness recruitment can be an important aspect of hyperacusis, the clinical diagnosis of hyperacusis in humans is mostly based on the functional and psychosocial impact of an abnormal sensitivity to sounds.^107^ Loudness recruitment may indeed be an important mechanism underlying decreasing loudness discomfort levels, but the relationship of loudness discomfort levels with hyperacusis is complex.^108^ Another reason to believe that the link between our findings and hyperacusis is still tentative at best is that as of yet there is no evidence that salicylate induces hyperacusis in humans.^5^

Even though the effects of salicylate on neuronal signaling have been intensely studied for >35 years, it is still far from clear how salicylate induces tinnitus. We showed some unexpected effects of salicylate on spontaneous and sound-evoked activity in the mouse IC. Both the lowering of spontaneous activity and the increased envelope encoding do not fit well with the induction of tinnitus by salicylate, suggesting that mice may not be the most suitable animals to study the mechanisms underlying salicylate-induced tinnitus. We suggest that the enhanced envelope coding can be expected to make an important contribution to the neural or behavioral effects of salicylate, and that our results thus contribute to the dissection of the complex effects of salicylate on the central auditory system.

### Limitations of the study

Our study has some limitations. We did not test for the presence or absence of behavioral evidence for salicylate-induced tinnitus or hyperacusis. The neurophysiological and behavioral effects of salicylate on AM encoding was not assessed at the same time. Only a single concentration of salicylate was employed, meaning that we cannot exclude that other results would have been found at a higher or a lower concentration. Since we only sampled the electrophysiological responses in the IC, we cannot exclude that the improved AM encoding is due to a central effect of salicylate. Our experiment did not allow us to identify whether a recorded neuron was excitatory and inhibitory, precluding the study of whether salicylate differentially affects the two types of neurons.

## STAR★Methods

### Key resources table

**Table:** 

REAGENT or RESOURCE	SOURCE	IDENTIFIER
**Bacterial and virus strains**		

AAV1.hSyn1.mRuby2.GSG.P2A.GCaMP6s.WPRE.SV40 from plasmid pAAV-hSyn1-mRuby2-GSG-P2A-GCaMP6s-WPRE-pA	Rose et al., 2016^109^	Penn Vector Core AV-1-50942; Now: Addgene viral prep # 50942-AAV1; RRID:Addgene_50942

**Chemicals, peptides, and recombinant proteins**		

3,3′-Dioctadecyloxacarbocyanine perchlorate (DIO)	Invitrogen	Cat#D275
Sodium salicylate	Sigma-Aldrich	Cat#31493

**Deposited data**		

Source code for figure generation and simulation	Zenodo/Github	https://doi.org/10.5281/zenodo.10843389 https://github.com/aaronbwong/salicylateonam
Source data for figures and analyses	Zenodo	https://doi.org/10.5281/zenodo.10843144

**Experimental models: Organisms/strains**		

Mouse: B6CBAF1/JRj	Janvier	N/A
Mouse: C57BL/6JRj	Janvier	N/A

**Software and algorithms**		

Igor Pro	WaveMetrics	RRID:SCR_000325
MATLAB	MathWorks	RRID:SCR_001622
SHARP-Track	Shamash et al., 2018^110^	https://github.com/cortex-lab/allenCCF
Kilosort2	Pachitariu et al., 2016^111^	https://github.com/MouseLand/Kilosort/releases/tag/v2.0
Phy2	Rossant et al., 2016^112^	https://github.com/cortex-lab/phy
R	CRAN	https://cran.r-project.org/
RHD USB Interface Board Software	Intan Technologies	RRID: SCR_019278

**Other**		

Multichannel silicon electrode	Cambridge Neurotech Ltd.	Cat#H3
RZ6 Multi-I/O Processor	Tucker Davis Technologies	RRID: SCR_018153
Intan USB interface board	Intan Technologies	Cat#C3100
RHD 64-channel headstage	Intan Technologies	Cat#C3315
Syringe infusion pump	Chemyx	Fusion 200

### Resource availability

#### Lead contact

Further information and requests for resources and reagents should be directed to and will be fulfilled by the lead contact, Gerard Borst (g.borst@erasmusmc.nl).

#### Materials availability

This study did not generate new unique reagents.

#### Data and code availability

• Curated and analyzed neurophysiology data reported in this paper has been deposited at Zenodo and is publicly available as of the date of publication. The DOI is listed in the key resources table. Additional data will be shared by the lead contact upon request.
• All code needed to generate the figures in this paper has been deposited at Zenodo and is publicly available as of the date of publication. The DOI is listed in the key resources table.
• Any additional information required to reanalyze the data reported in this paper is available from the lead contact upon request.

### Experimental model and study participant details

#### Animals

Adult male or female B6CBAF1/JRj mice, which are the F1 of a cross between C57BL/6JRj and CBA/JRj mice, were obtained from Janvier for behavior and acute electrophysiology recordings. We used n = 6 mice (4 males) for behavior; n = 10 (3 males) for the acute electrophysiology recordings, of which 4 (2 males) were from behavioral training. Adult male C57BL/6JRj mice (n = 2) were used for imaging experiments. Randomization and blinding was not performed, because each experiment utilized the experimental unit as its own control and therefore there were no separate treatment groups.

During the experimental period, the mice were housed with 2–4 mice per cage, enriched with a running wheel, an acrylic house and nesting material. Ethical approval was granted prior to the start of the experiments from the national authority (Centrale Commissie Dierproeven, The Hague, The Netherlands) as required by Dutch law, and all experiments were performed according to institutional, national, and European Union guidelines and legislation as overseen by the Animal Welfare Board of Erasmus MC.

#### Surgery

For mice used in behavioral experiments, a titanium head plate was attached to the skull of the animals for head fixation during the experiments. The animals were anesthetized through respiratory delivery of isoflurane, and maintained at surgical level of anesthesia, assessed through the hindlimb withdrawal reflex upon a toe pinch. A heating pad with a rectal feedback probe (40-90-8C; FHC, Bowdoinham, ME, USA) was used to maintain body core temperature at 36°C–37°C. Eye ointment (Duratears; Alcon Nederland, Gorinchem, The Netherlands) was used to keep the eyes moist during surgery. Boluses of buprenorphine (0.05 mg/kg; Temgesic, Merck Sharp & Dohme, Inc., Kenilworth, NJ, USA) and carprofen (5 mg/kg; Rimadyl, Zoetis, Capelle a/d IJssel, The Netherlands) were injected subcutaneously at the beginning of the surgery. The skin overlying the IC was incised. Lidocaine (Xylocaine 10%; AstraZeneca, Zoetermeer, The Netherlands) was applied before removing the periosteum and cleaning the skull. After etching the bone surface with phosphoric acid gel (Etch Rite; Pulpdent Corporation, Watertown, MA, USA), the titanium head plate was glued to the dorsal surface of the skull using dental adhesive (OptiBond FL; Kerr Italia S.r.l., Scafati, SA, Italy) and further secured with dental composite (Charisma; Heraeus Kulzer GmbH, Hanau, Germany).

For electrophysiology experiments, prior to headplate attachment as described above, anesthesia was induced by IP injection of a ketamine (Alfasan, Woerden, The Netherlands) and xylazine (Sedazine, AST Farma, Oudewater, The Netherlands) mixture (130 mg kg^−1^ and 13 mg kg^−1^, respectively). An intraperitoneal cannula (Butterfly Winged Infusion Set, 25G x ¾”, NIPRO EUROPE N.V., Zaventem, Belgium) allowed the injection of a maintenance solution of ketamine/xylazine with a syringe pump (Fusion 200, Chemyx, Stafford, TX, USA), which provided a continuous infusion rate of between 35 and 45 mg/kg/h of ketamine and 1.4–1.8 mg/kg/h of xylazine. The adequacy of anesthetic depth was evaluated by toe pinch, and the infusion rate was adjusted when needed. A second IP cannula was placed to facilitate systemic salicylate administration. Headplate attachment was performed as described above. A craniotomy of the skull overlying the left IC was performed for recording, and a second one overlying the left frontal lobe for the ground screw. To protect the craniotomy and minimize brain shift, the craniotomy was covered with 2% agarose dissolved in rat Ringer’s solution (148 mM NaCl, 5.4 mM KCl, 1 mM MgCl_2_, 1.8 mM CaCl_2_, 5 mM HEPES).

### Method details

#### Sound presentation

Sound stimuli were generated in MATLAB R2019a (The MathWorks, Natick, MA, USA) and played back via a TDT System3 setup (either a combination of an RX6 processor, PA5 attenuator and ED1 electrostatic speaker drivers, or an RZ6 processor) driving two ES1 electrostatic speakers (Tucker Davis Technologies, Alachua, FL, USA). Sound stimuli were presented bilaterally in the open field during behavioral experiments, and unilaterally to the right ear in the anesthetized experiments. Sound intensities were calibrated using a condenser microphone (ACO pacific Type 7016; ACO Pacific, Inc., Belmont, CA, USA) connected to a calibrated pre-amplifier and placed at the position of the pinnae.

The UM noise was a broadband pink (1/f) noise between 2 and 48 kHz. Each behavioral trial started with a UM noise with a 10 ms cosine squared ramp. After some time, the sound switched into a sinusoidally modulated version of the base stimulus according to:

$$y_{\mathrm{mod}}=y\times \left(1+m\times \sin\;\left(2\pi f_{\mathrm{mod}}t+\varphi \right)\;\right)$$

where y and y_mod_ are the base and amplitude-modulated (AM) stimuli, respectively; m is the modulation depth between 0 (no modulation) and 1 (full modulation); f_mod_ is the modulation frequency in Hz; t is time in s; and φ is an offset in phase to accommodate differences in instantaneous intensity (see below).

The SAM noise was designed to prevent detection of non-AM cues in the behavioral experiments.^49^ First of all, amplitude modulation creates sidebands and increases the overall intensity of the sound. The unmodulated part of each stimulus was generated by first creating another instance of the SAM noise of the trial, followed by randomization of the phase of the sideband in the spectral domain via Fourier transform. This created a UM noise which was matched to the SAM noise with respect to intensity and spectral properties. Secondly, to avoid an abrupt transition, the UM and SAM noise were cross-faded with a 20-ms window following a cosine and a sine function, respectively, between 0 and π/2. The Pythagorean identity sin^2^ θ + cos^2^ θ = 1 ensured that the combined instantaneous power of the two uncorrelated noises remained constant. Thirdly, the amplitude modulation started at the phase at which the instantaneous value of the sine function matched the average power of the UM noise: $\varphi =\arcsin\;\left(\sqrt{1+0.5\;m^{2}}-1\right)$, where m is the modulation depth.

For electrophysiology, SAM recording sets had a fixed UM duration of 1 s before transitioning into AM, which then played for another 1 s. The intertrial interval (ITI) was set to a minimum of 500 ms. AM parameters were: modulation frequency (16, 64, 128 & 512 Hz), modulation depth (0, 0.06, 0.125, 0.25, 0.5 & 1) and intensity (45 & 60 dB SPL). Trials were presented in pseudorandom order.

During electrophysiological experiments, pure-tone responses were recorded in FRA sets (frequency: 2–64 kHz in ¼ octave steps; intensity: 0–60 dB SPL in 5 dB steps; duration: 100 ms; intertrial interval: 200 ms; repetitions: 20, presented in pseudorandom order).

In order to assess the excitation-inhibition balance, a DRC stimulus was presented to construct an STRF. The DRC stimuli used in this study were similar to those in previous literature (e.g., Ref.^38^^,^^41^^,^^76^), except that the duration of the individual tones was variable. This is illustrated spectrographically in Figure S4A. Tone onsets, intensities and frequencies were randomized independently (5-ms bins in time, 1/12-octave bins covering 2–64 kHz and 5 dB bins covering 25–70 dB SPL). Tone durations varied between 20 ms and 200 ms (discretized in 5 ms steps) approximating an exponential probability distribution (λ = 30 ms), including 5-ms on- and off-ramps, each occupying a single time bin. The average tone density was two per octave. Each stimulus had a duration of ∼60 s, and was repeated 20 times without interruption.

#### Behavioral experiments

Head-fixed animals were trained to respond to the transition from UM to SAM noise by licking a water spout as reported in our previous study on AM detection.^49^ Briefly, the animals were motivated through water control: they received water only during training sessions and *ad libitum* water during the weekends (Friday evening to Sunday afternoon). To generalize the task, animals were trained at modulation frequencies between 4 and 1024 Hz in octave steps, modulation depths of 0.06, 0.125, 0.25, 0.5 and 1, and at intensities of 45 and 60 dB SPL. Trials that did not transition to AM were also presented, deemed catch trials, to assess the false alarm rate (FA).

To test the effect of salicylate on AM detection, an additional week of training was performed in combination with daily saline intraperitoneal injections to habituate them to the stress of receiving injections. This was followed by a test period consisting of three consecutive weeks: a baseline week, a salicylate week and a washout week. During this test period, only the modulation frequencies 16 and 512 Hz were presented to ensure that sufficient trials were acquired to estimate the AM-detection performance of the animals. On Mondays and Tuesday of each week, animals were trained to establish a stable level of motivation from water control. On Wednesdays through Fridays, either saline (baseline and washout weeks) or 250 mg/kg sodium salicylate (salicylate week) was injected intraperitoneally 2 h prior to the animal’s testing session. No testing was performed during the weekends.

The sensitivity measure d’ was calculated from the area under the curve (AUC) from receiver operating characteristic (ROC) analysis using the formula $d^{'}=\left|\sqrt{2}\;\Phi^{-1}\left(AUC\right)\right|$, where **φ**^−1^ represents the MATLAB function for the inverse cumulative distribution (‘norminv’). To avoid infinities in the d’ conversion, the behavioral measures (hit rates and false-alarm rates) were limited to 0.5/N_trial_ and 1-0.5/N_trial_, respectively.

The experimental unit for behavioral experiments was the animal (n = 6), with paired comparisons made between baseline and salicylate periods for each animal.

#### Two-photon imaging

We used ratiometric Ca^2+^ imaging to assess the effect of salicylate on the spontaneous firing rate of dorsal IC neurons. Recombinant AAV viral vector (200 nL; 1:10 diluted in PBS; AAV1.hSyn1.mRuby2.GSG.P2A.GCaMP6s.WPRE.SV40; titer after dilution: 1.08×10^12^ genome copies/mL; Penn Vector Core, University of Pennsylvania, Philadelphia, USA) was injected into the IC, 200 μm below the brain surface. The plasmid pAAV-hSyn1-mRuby2-GSG-P2A-GCaMP6s-WPRE-pA was a gift from Tobias Bonhoeffer & Mark Huebener & Tobias Rose (Addgene plasmid # 50942; http://n2t.net/addgene:50942; RRID:Addgene_50942). Infected neurons expressed both the green, Ca^2+^-sensitive GCaMP6s and the red, Ca^2+^-insensitive mRuby2.^109^ We used the ratio between green and red fluorescence as a measure for the spontaneous firing rate. Imaging experiments started 14 days after viral injections to allow sufficient time for the fluorescent proteins to express. We did not observe a significant change in mRuby2 fluorescence between successive sessions.

A custom built two photon microscope with a 20× water immersion objective (LUMPlanFI/IR, 20X, NA: 0.95; Olympus Corporation, Tokyo, Japan) was used for two-photon imaging via a cranial window.^113^ Excitation light was provided by a Mai Tai laser at a wavelength of 920 nm (Spectra Physics Lasers, Mountain View, CA, USA). Emitted light was separated by a dichroic mirror at 558 nm, a green bandpass filter centered at 510 nm (bandwidth: 84 nm; FF01-510/84-25; Semrock) and a red bandpass filter centered at 630 nm (bandwidth: 60 nm; D630/60; Chroma). Voltage gains of photomultiplier tubes were 700 V for both channels. Images were analyzed with custom procedures written in Igor Pro (WaveMetrics, Inc, Lake Oswego, OR, USA). Regions-of-interest (ROIs) were defined manually, encompassing the cell body of neurons. Only ROIs that were identified in baseline, salicylate and washout sessions were included in the final analysis. The average area of included ROIs was 237 ± 87 μm^2^. Raw fluorescence values were averaged across all pixels within an ROI during a silent period of around 10 min for both GCaMP6s (F_GCaMP_) and mRuby2 (F_mRuby_).

The experimental unit for imaging experiments were recorded neurons (n = 30, 2 mice) for which paired comparisons were made between baseline, salicylate and washout periods.

#### Electrophysiological recordings

A multichannel silicon electrode (64 channel, single linear shank, 1250 μm, H3, Cambridge Neurotech Ltd., Cambridge, United Kingdom) was coated with a saturated solution of DiO (3,3′-Dioctadecyloxacarbocyanine perchlorate, catalog number: D275, Invitrogen, ThermoFisher Scientific, Waltham, MA, USA) in 97% ethanol, and inserted under direct visual inspection during online monitoring of the channels for sound-evoked spikes. The electrode signals were digitized at a sampling frequency of 30 kHz at the headstage amplifier (RHD 64-channel headstage, part C3315, Intan Technologies), which was connected to an acquisition board (RHD USB-interface board, part C3100, Intan Technologies, Los Angeles, CA, United States) under the control of the RHD data acquisition GUI (Intan Technologies).

Prior to salicylate injection, baseline recordings were made of the FRA, SAM and DRC stimulus sets. Results from baseline FRA and SAM recordings were reported in a previous paper.^49^ Sodium salicylate (45 mg/mL, article number 31493, Sigma-Aldrich), was then injected intraperitoneally at a dose of 250 mg/kg through a cannula inserted into the peritoneal space. After salicylate injection, three additional SAM sets were recorded, the last of which was used as the salicylate reference set (corresponding to the 80–120 min post-salicylate period). After the third post-salicylate SAM set, an FRA and then a DRC set were recorded as well.

After completion of the recordings, the animals were perfused transcardially with 4% paraformaldehyde (PFA) in 0.12 M phosphate buffer under pentobarbital anesthesia. Before starting the perfusion, 0.2–0.5 mL of blood was collected by aspiration from the right ventricle using a 20G needle and placed in a K_2_EDTA tube (Microtainer, cat. number: 365974, BD, Franklin Lakes, NJ, USA) on ice. Tubes were centrifuged (3000 RPM, 4°C for 15 min^114^ to allow collection of plasma for the determination of the concentration of salicylate by enzyme immunoassay (Architect c4000, Abbott, Veenendaal, The Netherlands). After perfusion, the brain was taken out and postfixed in 4% PFA for 30 min. After storage in 10% sucrose, the brain was embedded in gelatine, sectioned on a freezing microtome and stained for DAPI. Images of slices (Axio Imager M2, Zeiss, Oberkochen, Germany) were used to reconstruct the location of the probe path within the IC, which was aligned to the Allen Brain Atlas Common Coordinate Framework v3 (CCFv3) using MATLAB code adapted from SHARP-Track.^110^ Using the coordinates of the tract and the location of single units along the linear electrode shank, the location of individual extracted units within the brain was determined.

Single-unit activity was obtained with spike sorting using Kilosort2^111^ followed by manual curation of the sorted results using Phy2 (https://github.com/cortex-lab/phy). The experimental unit for electrophysiology experiments were the single units (n = 55, 10 mice), with paired comparisons made between baseline and post-salicylate periods of each unit. Due to the highly heterogeneous patterns of activity of IC neurons and the uncertainty of the yield of units obtained per animal, no *a priori* calculation was deemed appropriate to estimate sample size. Single units whose activity (both spontaneous and evoked) was abolished after salicylate injection (n = 57) were excluded from analysis on the effect of salicylate, as no comparison could be made between their activity in the presence of salicylate compared to baseline.

### Quantification and statistical analysis

#### Pure-tone analysis

To map the tuning of single units to pure tones, the frequency autocorrelation areas (FACA; ref.^115^) were calculated from the kernel spike density (KSD) of the evoked spike rates. From this tuning curve, the characteristic frequency (CF, the frequency at which a significant response was produced at the lowest intensity) and the minimum threshold (MT, the lowest intensity at which a significant response was evoked) were calculated.

#### Analysis of electrophysiological responses to SAM noise

Temporal coding was quantified by a measure of neural synchronization to the envelope of AM, phase-projected vector strength (VS_PP_), as described in Yin et al.^116^ In contrast to traditional vector strength, VS_PP_ tolerates lower firing rates better. In order to eliminate onset effects, both VS and average firing rate were calculated from the spikes during the last 500 ms of the SAM stimulus.

One challenge in comparing inter-unit performance using the firing rate was the large variations in firing rates between units. Additionally, in order to compare the performance of AM encoding between the temporal and rate modalities, they had to be calculated on the same scale. To address these challenges, ROC analysis was performed to convert the calculated parameters to a performance score in d’ scale. A threshold was determined for each modulation frequency by finding the interpolated amplitude at which d’ crossed a value of 1.

We used a dimensionality reduction model to study the population activity of IC neurons.^49^ Briefly, spike times of all neurons (n = 55) were converted to trial-averaged peristimulus-time histograms (PSTHs; bin size: 3.9 ms). The difference between PSTHs averaged across all fully modulated stimuli and that averaged across all unmodulated stimuli in baseline condition were used as input to a principal component analysis (PCA). The largest resulting component was coined the “AM-encoding component” in this paper and was used in a subsequent simulation.

For simulating response latencies, single trial spike times were converted into kernel spike density (KSD) functions (Gaussian kernel, σ = 2 ms). Combined KSD functions were projected onto the AM-encoding component described above. Time for this component to cross an arbitrary “detection level” plus an additional 100–200 ms non-decision time was taken as the simulated response latency. As a goodness-of-fit measure, cumulative distribution functions (CDFs) of simulated response latencies, grouped by stimulus parameters, were compared to that of the behavioral data using their root-mean-square difference.

#### STRF estimation from DRC response

The analysis of responses to DRC stimuli followed Williamson et al*.*^41^ For each unit, we estimated how much of the total neuronal response power is predictable. We calculated the *signal power* according to Sahani and Linden:^74^

$$\text{signal}\;\text{power}:\hat{P}\left(\mu \right)=\frac{1}{N-1}\left(NP\left(\bar{r^{\left(n\right)}}\right)-\bar{P\left(r^{\left(n\right)}\right)}\right)$$

$$r^{\left(n\right)}=\mu +\eta^{\left(n\right)}$$

where N is the number of repetitions, $r^{\left(n\right)}$ is the response vector (PSTH) of the n^th^ trial, μ is the common, stimulus dependent component (signal) in the response and $\eta^{\left(n\right)}$ is the (zero-mean) noise component of the response in the n^th^ trial. “Power”, P(·), indicates averaged squared deviation from the mean over time. The bar $\bar{\cdot }$ indicates trial averages.

A similar estimator for *noise power* was obtained by subtracting $\hat{P}\left(\mu \right)$ from $\bar{P\left(r^{\left(n\right)}\right)}$. Only responses with a signal power of at least twice the noise power were included for further analysis.

STRFs (“simple model”) were then estimated using the automatic smoothness determination algorithm (ASD) algorithm.^54^ The contextual gain model (“context model”), was fitted using the alternative least square (ALS) approach of Ahrens et al*.*^75^ The modeled firing rate $\hat{r}$ at time i by the context gain model is given by the equation:

$$\hat{r}\left(i\right)=c+\sum_{j=0}^{J}\sum_{k=1}^{K}w_{j+1,k}^{tf}s\left(i-j,k\right)\left(1+\sum_{m=0}^{M}\sum_{n=-N}^{N}w_{m+1,n+N+1}^{\tau \varphi }s\left(i-j-m,k+n\right)\right)$$

where *s(t,f)* is the strength of the stimulus at time *t* and frequency bin *f*; the constant *c* sets a baseline firing rate; w^tf^ are weights associated with the STRF-like principal receptive field (PRF), defined in absolute frequency and time-lag preceding the response; w^τφ^ are weights associated with the context gain field (CGF), defined in terms of relative offsets of time (*τ*) and frequency (*φ*).

Each least-squares step was regularized using either the ASD-derived optimal spectrotemporal smoothing (for the PRF) or a fixed smoothing bandwidth of 2.5 ms (0.5 bin) and 1/6 octave (for the CGF). The models were compared by calculating their predictive power and normalizing to the predictable signal power as calculated above:^74^

$$\text{normalized}\;\text{predictive}\;\text{power}:\frac{P\left(r\right)\;–\;P\left(r\;–\;\rho \right)}{\hat{P}\left(\mu \right)}$$

where ρ is the predicted response. Thus, the predictive power is the difference between the power of the observed response P(r) and the power of the residual P(r–ρ) (“error power”).

This procedure was performed with cross-validation, thus measuring the generalization performance of the model and providing a lower estimate of the predictive power.^41^^,^^74^ Generalization performance was assessed using 10-fold cross-validation, reserving a randomly distributed disjoint subset of one-tenth of the bins as the validation set for each of the twenty repetitions.

To investigate input linearity, s(t,f) was defined using different lookup scales. The highest intensity (70 dB SPL) was defined as 1. The power (“pow”) and amplitude (“amp”) scales followed the definition of the decibel scale with s(t,f) = 10ˆ((x(t,f) - 70)/10) and s(t,f) = 10ˆ((x(t,f) - 70)/20), respectively, where x(t,f) is the intensity in dB SPL. The dB0, dB20 and dB40 were defined directly in decibels with the reference values of 0 dB, 20 dB and 40 dB, respectively, where:

s(t,f) = x/(70 - ref) for x > ref; s(t,f) = 0 for x ≤ ref; for the binary scale, all bins with a sound intensity were given the value of 1.

#### Statistical analysis

A t-test was used to compare means, unless data were not normally distributed. In that case a Wilcoxon rank-sum test was used. *p*-values were Bonferroni-corrected for multiple comparisons.

The effect of salicylate on d’ was tested with a series of linear mixed-effect models on a subset of data with specific modulation frequency and sound intensity. These models were implemented with the lme function from the nlme package in R, version 3.1–153; https://CRAN.R-project.org/package=nlme.^117^ In each model, d’ (“dPrime”) was modeled with modulation depth (“modDepth”) and its interaction with treatment conditions (“modDepth:condition”) as fixed effects. Training batch (“batch”) and mouse (“subject”) were used as hierarchical random effects. Formula: dPrime ∼ modDepth + modDepth:condition + (1|batch/subject). Significant differences were determined using *p*-values associated with the coefficients of the interaction terms (modDepth:condition). The effect of salicylate on thresholds was tested with a linear mixed-effect model implemented with the lmer function from the lme4 package in R, version 1.1–28; https://CRAN.R-project.org/package=lme4;^118^. Threshold (in dB) was modeled with stimulus intensity (“intensity”), modulation frequency (“frequency”) and treatment condition (“condition”) as fixed effects, all as categorical factors with all possible interactions; training batch (“batch”) and mouse (“subject”) were used as hierarchical random effects. Formula: threshold ∼ frequency∗intensity∗condition + (1|batch/subject). Subsequently, a total of six specific hypotheses were tested on the model using general linear hypothesis testing implemented in the glht function in the R package multcomp version 1.4–18; https://CRAN.R-project.org/package=multcomp.^119^
