# Supplementary material for: Sodium salicylate improves detection of amplitude-modulated sound in mice
Source: iScience. 2024 Apr 9;27(5):109691. doi: 10.1016/j.isci.2024.109691 (PMC11088340; doi:10.1016/j.isci.2024.109691)
Supplement: Document S1. Figures S1–S4 [file mmc1.pdf]

iScience, Volume *27*

Supplemental information

Sodium salicylate improves detection of amplitude-modulated sound in mice

Maurits M. van den Berg, Aaron B. Wong, Ghais Houtak, Ross S. Williamson, and J. Gerard G. Borst

Supplementary Figures

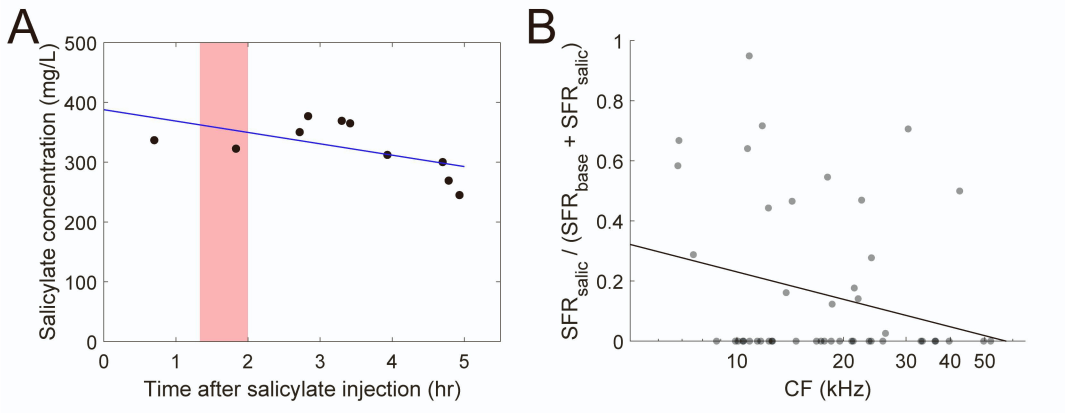

Figure S1. Changes in plasma salicylate concentration over time from blood samples obtained at the end of an experiment and their impact on the spontaneous firing rate, related to Figure 1 and STAR Methods. A) Linear fit of the time-dependent changes in the plasma salicylate concentration follows the equation: $C = -19\,t + 388$, where t is the time since salicylate injection (in hours) and C is the plasma salicylate concentration in mg/L. Salicylate concentration towards the end of the reference recording of AM (red shaded field) is about 350 mg/L. B) The reduction in spontaneous firing rate by salicylate can be observed at a large range of CFs. Relation between the relative reduction in spontaneous firing rate in the presence of salicylate and the CF of the unit. A value of 0.5 indicates no change. Solid line is the regression line, whose slope did not show a significant relationship between the change in spontaneous firing rate and CF.

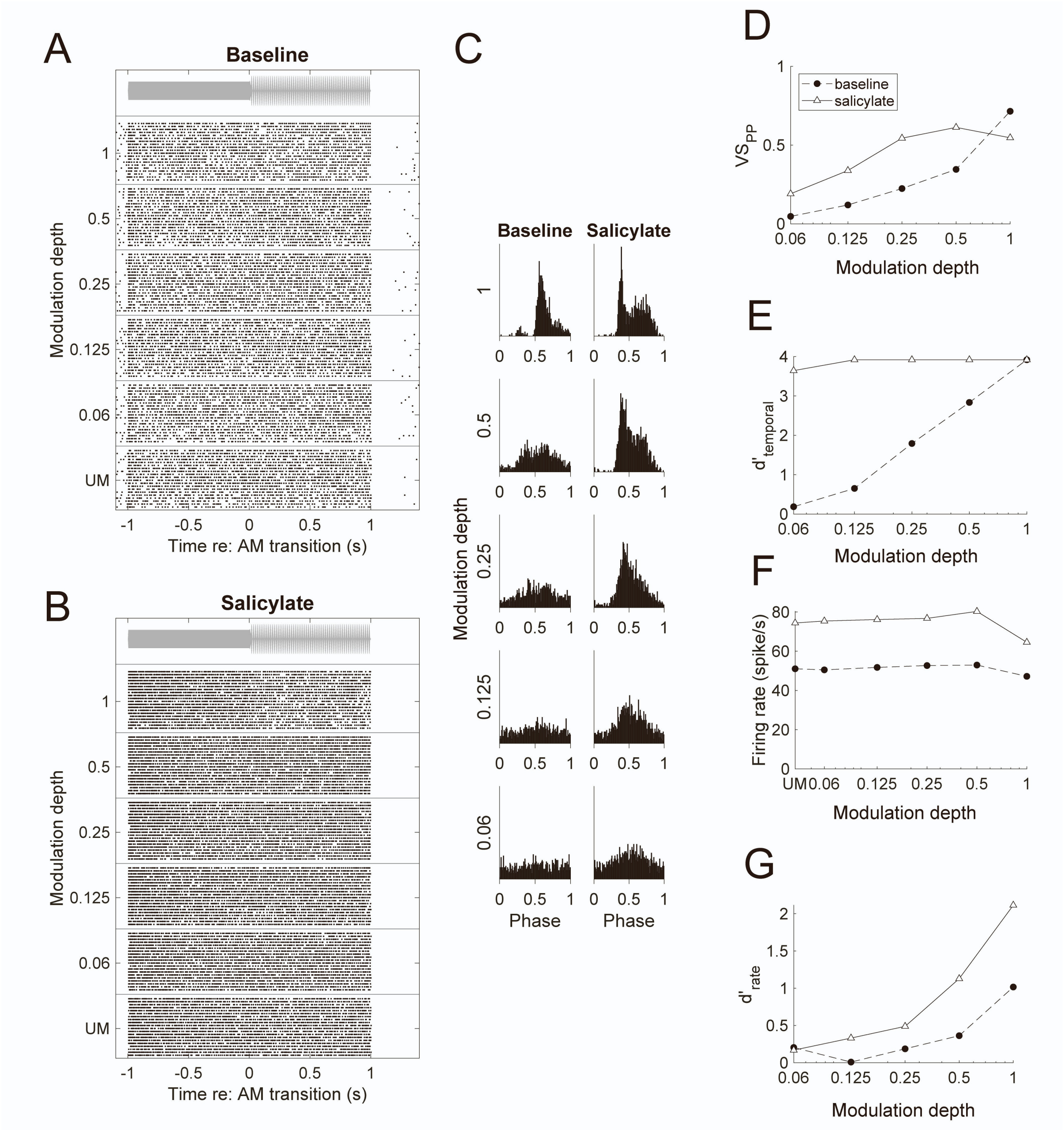

Figure S2. Salicylate improved phase locking to SAM noise, related to Figure 3. Dot raster plots of the response to 60 dB SPL, 64 Hz SAM noise during baseline (A) and in the presence of salicylate (B), with the trials sorted by decreasing modulation depth. UM noise started at -1 s. Stimulus envelope is shown at the top of the panels. (C) Cycle histograms show improved phase-locking of spikes to the modulation period in the presence of salicylate. Full y-axis scale is 0.1 spike/cycle/bin; bin size is 0.13 ms. The effect of salicylate on temporal coding of AM is shown by (D) VS_{PP}, and I d’ calculated from VS_{PP}, while its effect on rate coding is shown in (F) for the raw firing rate and (G) for the calculated d’.

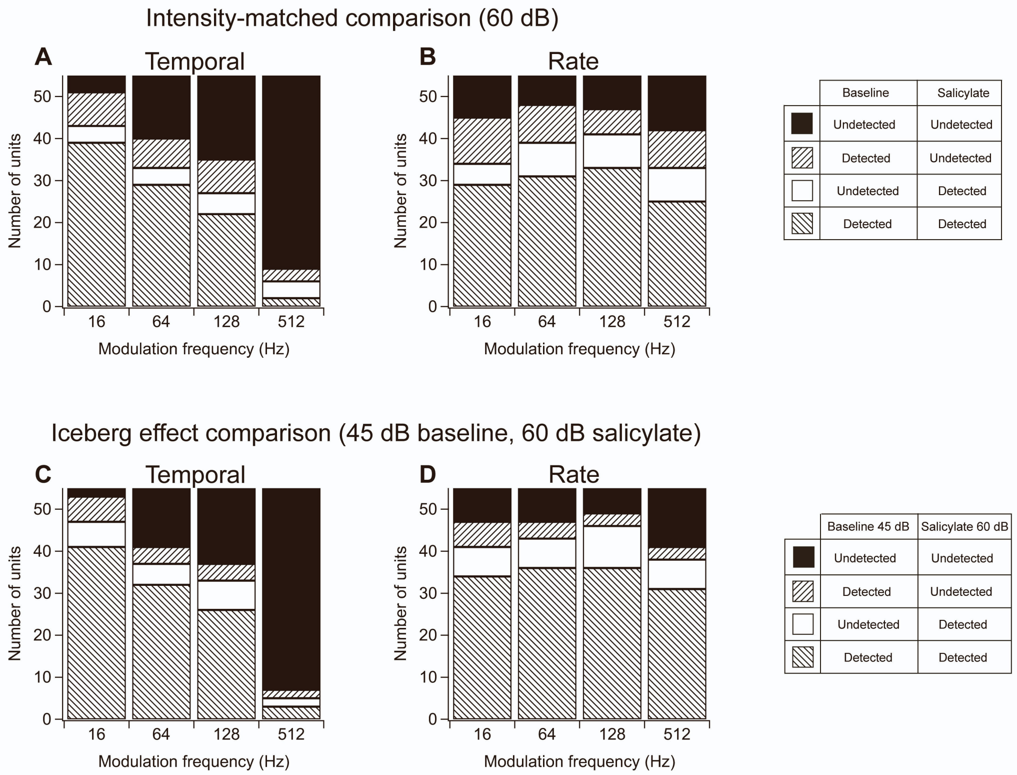

Figure S3. Unit detection behaviour after salicylate. A-B) The intensity matched comparison shows a lack of systematic change in AM detectability after application of salicylate, related to Figure 4. A) For each unit at the different modulation depths of 60 dB SPL stimuli, VS_{PP} was converted to a sensitivity index ($d'_{temporal}$), and a threshold was defined as the modulation depth where $d'_{temporal}$ equalled 1. B) as A) but for d'_{rate} thresholds (based on rate instead of VS_{PP}). C-D) To test the iceberg effect hypothesis: for each unit, it was compared whether a threshold for $d'_{temporal}$ could be calculated both for 45 dB SPL stimuli under control conditions and for 60 dB SPL stimuli in the presence of salicylate, related to Figure 5. Each stacked bar graph show for the different modulation frequencies from bottom to top the number of units with a detection threshold both before and in the presence of salicylate, a threshold only in salicylate, a threshold only in baseline conditions, and no threshold during both conditions.

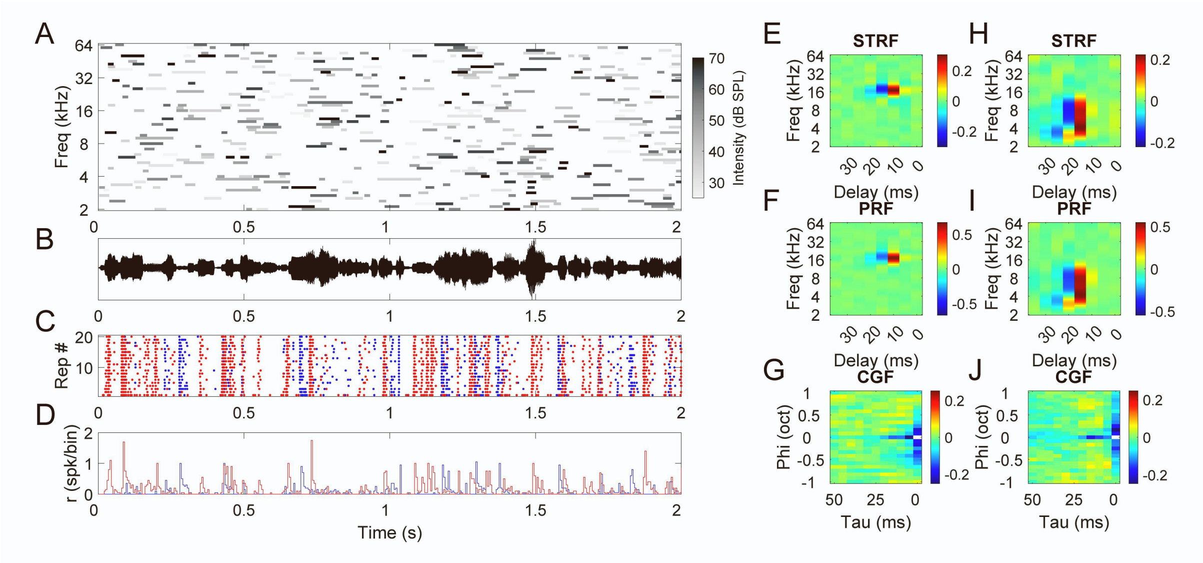

Figure S4. Using a DRC stimulus to measure the STRF of IC neurons, related to Figures 5-6. A) spectrogram showing the first 2 seconds of an example DRC stimulus, composed of tones with randomized onset times, intensities and durations. B) Waveform of stimulus in A). C) Raster plot of spike times from example IC units A and B (blue and red, respectively) to 20 repetitions (‘Rep #’) of the DRC stimulus shown in A and B. D) Average peristimulus time histogram at 5-ms time bins of the responses in C. E) STRF of example unit A (blue in C-D) showing a BF of about 16 kHz. F) Principal receptive field (PRF) of unit 1. G) Context gain field (CGF) of unit A. H) STRF of unit B (red in C-D) showing broad tuning between 3-12 kHz. I) PRF of unit B. J) CGF of unit B.
